# Boosting Electrochemical Carbon Dioxide Reduction on Atomically Dispersed Nickel Catalyst

**DOI:** 10.3389/fchem.2021.837580

**Published:** 2022-01-20

**Authors:** Qi Hao, Dong-Xue Liu, Ruiping Deng, Hai-Xia Zhong

**Affiliations:** ^1^ Key Laboratory of Automobile Materials, Ministry of Education, School of Materials Science and Engineering, Jilin University, Changchun, China; ^2^ State Key Laboratory of Rare Earth Resource Utilization, Changchun Institute of Applied Chemistry, Chinese Academy of Sciences, Changchun, China; ^3^ Center for Advancing Electronics Dresden (cfaed), Faculty of Chemistry and Food Chemistry, Technische Universität Dresden, Dresden, Germany

**Keywords:** ligand-mediated, atomic dispersion, nickel clusters and single atoms, nickel-nitrogen catalytic sites, carbon dioxide reduction

## Abstract

Single-atom catalysts (SACs) with metal–nitrogen (M–N) sites are one of the most promising electrocatalysts for electrochemical carbon dioxide reduction (ECO_2_R). However, challenges in simultaneously enhancing the activity and selectivity greatly limit the efficiency of ECO_2_R due to the improper interaction of reactants/intermediates on these catalytic sites. Herein, we report a carbon-based nickel (Ni) cluster catalyst containing both single-atom and cluster sites (NiNx-T, T = 500–800) through a ligand-mediated method and realize a highly active and selective electrocatalytic CO_2_R process. The catalytic performance can be regulated by the dispersion of Ni–N species *via* controlling the pyrolysis condition. Benefitting from the synergistic effect of pyrrolic-nitrogen coordinated Ni single-atom and cluster sites, NiNx-600 exhibits a satisfying catalytic performance, including a high partial current density of 61.85 mA cm^−2^ and a high turnover frequency (TOF) of 7,291 h^−1^ at −1.2 V vs. RHE, and almost 100% selectivity toward carbon monoxide (CO) production, as well as good stability under 10 h of continuous electrolysis. This work discloses the significant role of regulating the coordination environment of the transition metal sites and the synergistic effect between the isolated single-site and cluster site in enhancing the ECO_2_R performance.

## Introduction

Electrochemical carbon dioxide reduction (ECO_2_R) of valuable fuel/chemicals, driven by renewable energy sources, offers a promising route to solve global warming and realize carbon neutrality (Shaikh et al., 2018; Sharifian et al., 2021). In particular, the produced high value-added chemicals/fuels can contribute to an industrial production-oriented carbon cycle and greatly alleviate the energy crisis (Kondratenko et al., 2013; Qiao et al., 2014). So far, a variety of catalysts (metal-free and metal-based catalysts, metal/covalent organic frameworks catalysts, etc.) have been widely developed to boost ECO_2_R of the target products with high activity and selectivity in order to achieve a high conversion efficiency (Duan et al., 2017; Vasileff et al., 2018; Liu et al., 2018). Currently, noble metal catalysts, such as Au and Ag, have demonstrated a promising catalytic performance concerning the generated current density (>10 mA cm^−2^) and Faradaic efficiency (>90%) for CO products, offering feasibility for CO_2_ valorization (Ma et al., 2016; Wuttig et al., 2016). However, their high cost seriously limits further application of these noble metal catalysts in ECO_2_R. To address these challenges, exploring carbon-based metal–nitrogen (M–N) catalysts with atomically dispersed transition metal sites would be a compelling choice due to their low cost, tunable structure, considerable activity, good stability, etc. Moreover, their well-defined coordination environment facilitates the clarification of the active sites and catalytic mechanism, as well as deeper investigation of the local microenvironmental effects on the activity (Li Y et al., 2021; Chen et al., 2021; Lu et al., 2021).

Transitional M–N-based single-atom catalysts (SACs) have emerged as one of the novel class of carbon-based catalysts to replace the noble metal catalysts (Chen et al., 2021). This is because of their ability to largely facilitate the ECO_2_R process while inhibiting the competing hydrogen evolution reaction (HER) by the virtue of their unique electronic structure, tailorable structure, and maximum atom utilization (Yang Q et al., 2019). Given that ECO_2_R is a thermodynamically controlled reaction process, SACs equally encounter some inherent drawbacks that mainly limit their practical development. For example, Fe/Co SACs with uniform Fe-N/Co-N sites have an ultralow overpotential for converting CO_2_ to CO. However, the product selectivity and current density of these catalysts at relatively high potentials will rapidly decrease due to the high energy barrier of CO desorption and the blockage of the active sites (Lin et al., 2019). In another case, despite a high CO selectivity, Ni SACs with Ni–N active sites often need a high overpotential to acquire considerable current density due to the weak adsorption of *COOH intermediates and thus low efficiency for the following *COOH intermediates generation (Ding et al., 2021). Therefore, it is imperative to develop SACs with optimized active centers that can exhibit a moderate interaction with each intermediate including *COOH and *CO toward promoting electrocatalytic ECO_2_R at low overpotential with high selectivity (Shi et al., 2014).

Herein, we develop atomically dispersed Ni-cluster catalysts with Ni–N coordination (NiNx-T, T = 500–800) through a ligand-mediated pyrolysis method. The resultant NiNx-T possesses well-defined isolated single-atom and sub-nanometer-sized cluster Ni sites with a pyrrolic-N coordination environment. By regulating the content of Ni-pyrrolic N and the dispersion of Ni species *via* controlling the pyrolysis condition, the ECO_2_R performance of NiNx-T can be optimized. The electrochemical characterizations show that NiNx-600 with optimized Ni-cluster exhibits higher catalytic activity than other contrast samples and Ni nanoparticles (Ni NPs), including a higher current density of 61.85 mA cm^−2^, faster reaction rate with TOF of 7,291 h^−1^, as well as more impressive selectivity of nearly 100% toward CO production. It also demonstrates a good stability over 10 h of continuous operation. This work highlights the significant role of regulating the coordination of transition metal sites and the synergistic effect between the isolated single-site and cluster site in enhancing the ECO_2_R performance.

## Materials and Methods

### Chemicals

Nickel (II) acetate tetrahydrate [Ni (Ac)_2_·4H_2_O, 99%] and 1,4,7,10-tetraazacyclododecane (cyclen, 97%) were purchased from Aladdin. XC-72R was purchased from SCI Materials Hub. Ultrapure water was used in all experiments [Millipore, 18.2 MΩ·cm]. All chemical reagents were used as received without further purification.

### Synthesis of Carbon-Based Ni-Cluster Catalysts

In a typical synthesis, Ni (Ac)_2_·4H_2_O (8.5 mg) and cyclen (18.5 mg) were dissolved in 1.5 ml of water and then stirred for 1 h at ambient conditions to obtain the violet solution. Subsequently, 100 mg of the XC-72R carbon carrier was added to the solution and stirred for 6 h. The resulting mixture was freeze-dried at −60°C for 12 h to obtain the black powder. Next, the powder was transferred into a porcelain dish and heated at 600°C, under argon (Ar) atmosphere. After cooling down to room temperature, the product was washed with ultrapure water and ethanol several times and dried in an oven at 70°C for 12 h (named NiNx-600). Other samples were synthesized *via* a similar procedure, except changing the pyrolysis temperature (500, 700 and 800°C, named NiNx-500, NiNx-700, and NiNx-800).

### Synthesis of Ni NPs

The Ni NPs were obtained *via* a similar method using NiNx-600, in addition to replacement of pyrolysis atmosphere (Ar) with 5% H_2_/Ar.

### Characterizations

Powder X-ray diffraction (PXRD) patterns were collected using an X-ray diffractometer (Rigaku) with Cu Kα radiation (λ = 0.15406 nm). Scanning electron microscope (SEM) images were acquired from a Hitachi S4800 field emission electron microscope (10 kV). Transmission electron microscope images and corresponding energy-dispersive X-ray (EDX) elemental mapping were obtained using JEM-2100F and FEI Talos F200x electron microscopes (200 kV). High-angle annular dark-field STEM (HAADF-STEM) images were obtained from an FEI Themis Z STEM with double spherical aberration correctors working at 300 kV. Raman spectra were collected using a micro-Raman spectrometer (Renishaw). N_2_ absorption/desorption isotherms were recorded on a Quantachrome Nova station. The X-ray photoelectron spectroscopy (XPS) analysis was carried out using a ThermoFisher Scientific ESCALAB 250Xi spectrometer. All spectra were calibrated according to the C 1s binding energy at 284.4 eV. The Ni loading of catalysts was conducted on an inductively coupled plasma-optical emission spectroscope (ICP-OES, PE Avio 200).

### Electrochemical Measurements

The evaluation of ECO_2_R performance was carried out in a classical three-electrode H-type system with a Nafion 212 film as separated component. An Ag/AgCl electrode was applied as a reference electrode and Pt mesh was used as a counter electrode. A carbon paper (Ce Tech Co. Ltd., N1S1007) was used as a working electrode. In a typical electrode preparation, the catalyst (5 mg) was dispersed in a mixed solution of ethanol (200 μL) and 0.5% Nafion solution (50 μL) by continuously sonicating for 1 h, and the dispersion liquid was drop-casted on a carbon paper with a certain catalyst loading of 0.5 mg cm^−2^. The geometric surface area of the electrode was applied to calculate the current density. All the applied potentials were changed to a reversible hydrogen electrode (RHE) based on the following equation:

$$\begin{array}{c} E_{RHE}=E_{Ag/AgCl}+0.05916\times pH+0.197 \end{array}$$ (1)

Electrochemical measurements were processed on a Biologic VMP3 electrochemical workstation at ambient conditions. Linear sweep voltammetry (LSV) curves were acquired with a scan rate of 10 mV s^−1^ in the Ar-and CO_2_-saturated 0.5 M KHCO_3_ solution. Electrochemical impedance spectroscopy (EIS) was carried out in the frequency range of 100 kHz to 0.1 Hz. For estimating the electrochemically active surface area, cyclic voltammetry (CV) curves were obtained from 20 to 120 mV s^−1^with an interval of 20 mV s^−1^ at the potential range of 0.1–0.2 V vs. RHE. Chronoamperometry (CA) was measured at various potentials with a fixed time of 20 min, and the gas-phase components were detected *via* gas chromatography equipped with PDD and FID detectors (ThermoFisher Scientific, Trace1300). High purity helium (99.9999%) and nitrogen (99.9999%) were employed as a carrier and make-up gas for chromatography, respectively. The liquid-phase products were analyzed using a Bruker NMR spectrometer (AVANCE-III HD 500). The FE of CO (FE_CO_) and TOF were calculated *via* the following equations:

$$\begin{array}{c} FE_{\mathrm{CO}}=\frac{Q_{\mathrm{CO}}}{Q_{\mathrm{Total}}}=\frac{Z\times n\times F}{Q_{\mathrm{total}}} \end{array}$$ (2)

$$\begin{array}{c} TOF=\frac{\frac{J_{\mathrm{product}}}{\mathrm{nF}}}{\frac{m_{\mathrm{cat}\times \omega }}{M_{\mathrm{Ni}}}} \end{array}$$ (3)

where Q is the total charge transferred through the working electrode at different potentials, Z is the number of electrons transferred, which is 2 for both CO and H_2_, n is the number of moles for a certain product, F is the Faradaic constant (96,485 C mol^−1^), J_product_ is the partial current density of a certain product, m_cat_ is the catalyst loading, ω is the Ni content of the catalyst, and M_Ni_ is the atomic mass of Ni (58.6934 g mol^−1^).

## Results and Discussions

**FIGURE 1:**
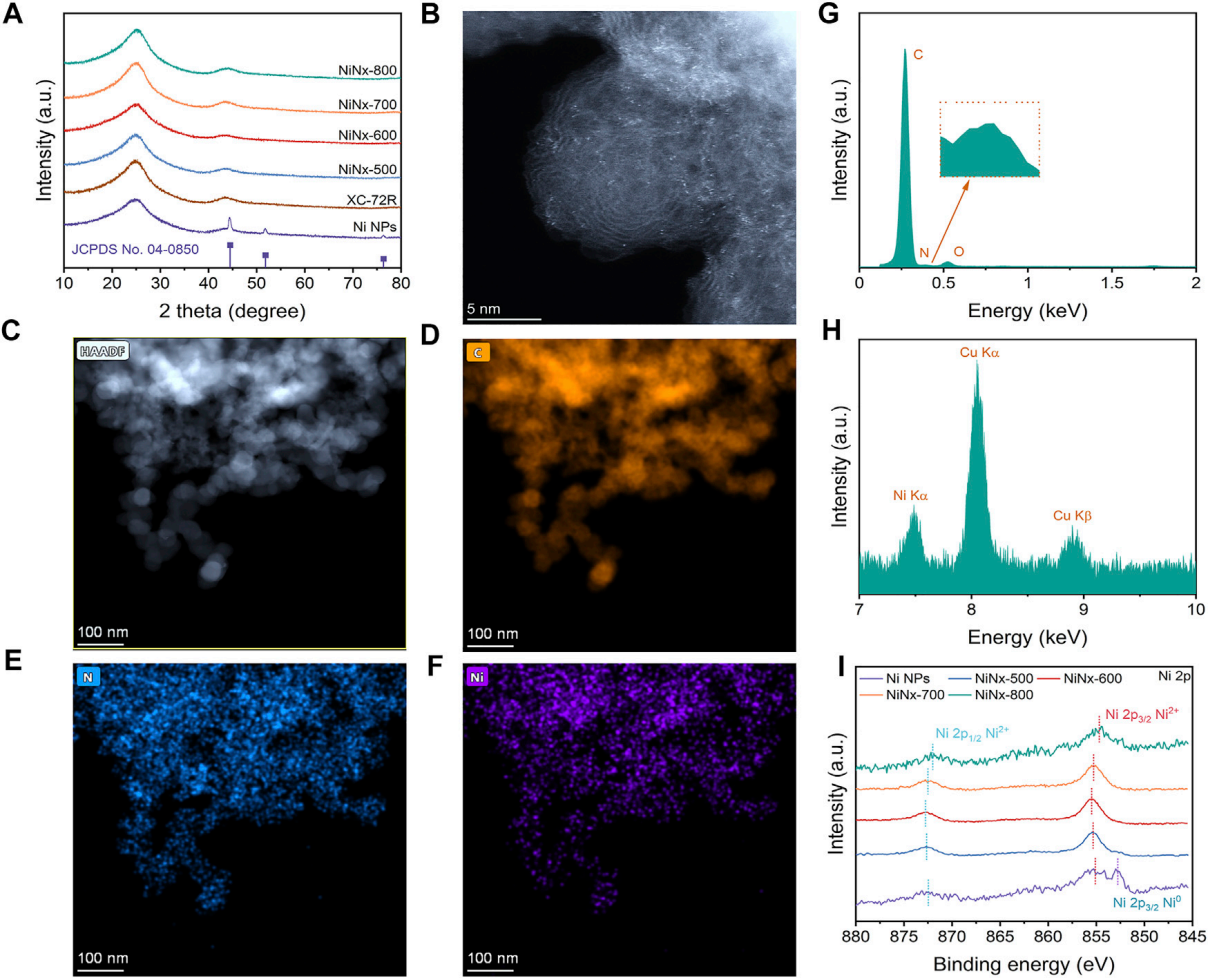
**(A)** XRD patterns of Ni NPs, XC-72R, and NiNx-Ts, respectively. JCPDS profile refers to metallic nickel (04–0,850). **(B)** HAADF-STEM image of NiNx-600. **(C)** Low-resolution HAADF-STEM image of NiNx-600. **(D–F)** STEM-EDX mapping of NiNx-600. **(G,H)** STEM-EDX analysis of NiNx-600. **(I)** High-resolution Ni 2p XPS spectra of Ni NPs and NiNx-Ts, respectively.

The synthesis process is shown in Supplementary Figure S1. In the process, through a ligand-mediated strategy, Ni ions could be coordinated with cyclen to form a Ni–cyclen complex and transformed to Ni–N species during pyrolysis. Figure 1A shows the PXRD patterns of the as-prepared catalysts, in which three diffraction peaks at 44.5°, 51.8°, and 76.4° were observed for Ni NPs, separately assigned to (111), (200), and (220) planes of crystalline Ni species (Ren et al., 2019). Only two broad graphitic peaks at 22.1° and 44.1° appear in the XRD patterns of NiNx-500 to NiNx-800 samples, which is similar to the XC-72R carrier and could be attributed to (002) and (100) planes of carbon (Yang H et al., 2019). The annular patterns in selected area electron diffraction (SAED) (Supplementary Figures S2–S6) further confirm the absence of crystalline Ni species (Zhang E et al., 2019). Moreover, none of the lattice stripes belonging to crystalline nickel species were detected in the high-resolution TEM (HRTEM) images of NiNx-500 to NiNx-800. These results all together confirm the amorphous state of Ni species on the carrier and verify the absence of Ni NPs for NiNx-T samples (Yan et al., 2018). On the contrary, the TEM and HRTEM images show the presence of Ni nanoparticles with a diameter of approximately 50 nm, accompanied with the exposed (111) plane (Fan et al., 2020), which is consistent with its XRD pattern (Supplementary Figures S8A–D). The corresponding EDX mapping of Ni NPs illustrates the dispersion of Ni and N elements on the carbon carrier, suggesting the loading of N-doped Ni NPs on carbon substrate (Supplementary Figures S8E–G). The loading of Ni is 1.77, 0.60, 0.52, 0.46, and 0.13 wt.% for Ni NPs and NiNx-T, respectively, as indicated by the ICP-OES measurement (Supplementary Table S2). The decrease in the nickel species content is due to volatilization of metal species during high-temperature pyrolysis. To accurately verify the dispersion of Ni species in NiNx-500 to NiNx-800, HAADF-STEM measurements were carried out. As shown in Figure 1B, plenty of bright dots can be observed as isolated Ni single-atom and Ni clusters on the carrier for NiNx-600 (Wan et al., 2020). In addition, the relevant EDX elemental mapping and analysis (Figures 1C–E) disclose the homogeneous distribution of Ni, C, and N elements for NiNx-600, which illustrate the uniform dispersion of Ni species on the carbon carrier. Interestingly, when the temperature increases from 500 to 600°C, the dispersion of Ni atoms increases, and thus, the agglomeration of Ni species is reduced due to a thermal migration process. However, further increase in the temperature from 600 to 800°C results in a significant increase in the degree of agglomeration of Ni species owing to the unavoidable Ostwald ripening (Supplementary Figures S9–S11) (Wang et al., 2018). Therefore, the pyrolysis temperature plays a crucial role in controlling the dispersion of Ni species.

To investigate the surface area and pore structure, nitrogen adsorption and desorption isotherms were acquired at 77.3K. As shown in Supplementary Figure S15, the corresponding pore size distributions show the presence of a mesoporous carbon skeleton for NiNx-T samples. It is thus clear that the carbon carrier has a good structural stability to ensure the sufficient exposure of abundant active sites during the catalytic process (Tan et al., 2018). In addition, compared with the XC-72R carbon carrier, the Brunauer–Emmett–Teller (BET) surface area of the NiNx-T samples (190.0, 142.0, 128.4, and 116.8 m^2^ g^−1^, respectively) loaded with Ni species is lower, which is attributed to the possible collapse of the pore structure during secondary pyrolysis and the import of Ni species with higher atomic mass than the C atom (Lin et al., 2019). Raman spectra in Supplementary Figure S14 show that the proportion of the D and G band (I_D_/I_G_; D: disorder, G: graphite) for NiNx-T is higher than that of the XC-72R carbon support, suggesting more local defects were introduced on the carbon carrier after secondary pyrolysis (Zhang E et al., 2019). Moreover, the amounts of defects for NiNx-T samples increase along with rising the pyrolysis temperature from 500 to 700°C. When pyrolyzed at 800°C, the I_D_/I_G_ value of NiNx-800 was reduced due to the increased degree of graphitization at higher temperatures. Thus, optimized pyrolysis conditions are beneficial for affording abundant pores and defects, facilitating the anchoring of Ni atoms toward the uniform dispersion of Ni species, which is favorable for sufficient mass/electron transfer during the catalytic process.

To investigate the chemical state and coordination environment, the X-ray photoelectron spectroscopy (XPS) technique was implemented. The high-resolution Ni 2p XPS spectrum (Figure 1I) of Ni NPs exhibit three peaks at 852.8, 855.5, and 873.1 eV, respectively, which are ascribed to Ni 2p_3/2_ Ni^0^, Ni 2p_3/2_ Ni^2+^, and Ni 2p_1/2_ Ni^2+^, respectively, indicating that the average chemical state of the nickel species in Ni NPs is between 0 and +2 (Feng et al., 2021). Unlike Ni NPs, no peaks attributed to Ni2p_3/2_ Ni^0^ appears in the high-resolution Ni 2p XPS spectra of NiNx-500 to NiNx-700. Only two peaks, which belong to Ni 2p_3/2_ Ni^2+^ and Ni 2p_1/2_ Ni^2+^, appear at binding energy at 852.8 and 873.1 eV, respectively. The higher binding energy of Ni 2p_3/2_ Ni^2+^ in the NiNx-600 samples reveal the higher oxidation state of Ni species compared to that in other NiNx-T samples (Jia et al., 2021). This is due to the increased concentration of dispersed Ni–N species along with the reduced amounts of Ni clusters by the optimization in metal dispersion, which is consistent with the aforementioned HAADF-STEM results. The high-resolution C 1s spectra can be deconvoluted into four peaks located at 284.4, 286.2, 289.04, and 290.86 eV, respectively (Supplementary Figure S12), which are attributed to C=C, C-O/C-N, C=O, and the satellite peak of C 1s, respectively (Hou et al., 2019). The high-resolution N 1 s spectra of the samples indicate the presence of pyridinic N (398.1 eV), pyrrolic N (399.3 eV), graphitic N (400.5 eV), and quaternary N (401.37 eV) species (Supplementary Figure S13) for NiNx-T samples (Cheng et al., 2018). Notably, apart from Ni NPs, the peak at 398.8 eV attributed to Ni–N coordination was observed for NiNx-T samples, stemming from the pyrolytic Ni–cyclen complex precursors, in which Ni was coordinated to N atoms in a five-membered ring. Moreover, the higher amount of pyrrolic N species than pyridinic N moieties also implies that most of the Ni atoms on the carrier are coordinated with pyrrolic N instead of pyridinic N (Li J et al., 2021). NiNx-600 has an optimized Ni–N content, outperforming other samples. The content of Ni–N species shows a volcanic trend when increasing the pyrolysis temperature and reaches a maximum value at 600°C, suggesting that Ni atoms are gradually coordinating with N and resulting in the atomic dispersion of the Ni site at higher temperatures.

**FIGURE 2:**
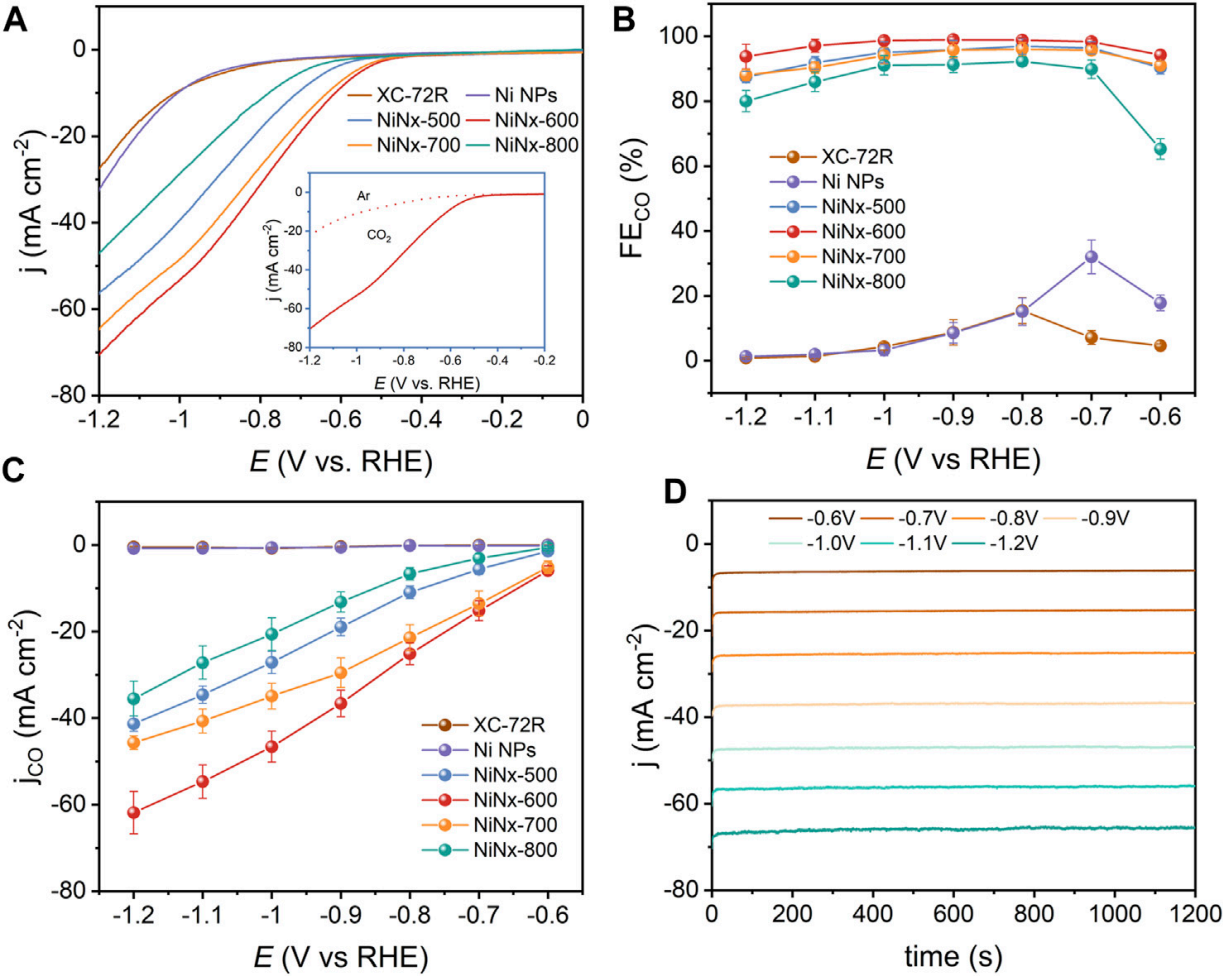
**(A)** LSV curves in Ar- and CO_2_-saturated 0.5 M KHCO_3_. **(B)** FEs of CO. **(C)** Partial current densities of CO for different catalysts and **(D)** CA curves of NiNx-600 at different applied potentials.

The evaluation of ECO_2_R activities of XC-72R, Ni NPs, and NiNx-Ts were measured in a typical three-electrode H-type cell containing a 0.5 M KHCO_3_ electrolyte (Supplementary Figure S16). As shown by LSV curves in Figure 2A, NiNx-600 exhibits a higher current density and lower onset potential in CO_2_-saturated electrolyte from −0.5 to −1.2 V vs. RHE, which observably exceeds with those of the other five samples. Moreover, for NiNx-600, the current density significantly decreases and the onset potential raises when the electrolyte is changed from CO_2_-saturated to Ar-saturated solution, which suggests its superiority for ECO_2_R to the competitive HER process and manifests that the boosted activity of NiNx-600 originates from CO_2_ reduction (Zhang H et al., 2019). Chronoamperometry (CA) was used to evaluate the total current density. Meanwhile, the gas products were detected by online GC (Figure 4B), and the liquid products were analyzed by offline 1H NMR (Supplementary Figure S19). The results indicate that only H_2_ and CO are detected for the synthesized samples during the ECO_2_R process. Figure 2B and Supplementary Figure S17 demonstrate the CA curves from −0.6 to −1.2 V vs. RHE. NiNx-600 holds the highest total current density with current densities of −6.25 mA cm^−2^ at −0.6 V vs. RHE and −65.93 mA cm^−2^ at −1.2 V vs. RHE, surpassing those of NiNx-500 (−1.58 and −47.24 mA cm^−2^), NiNx-700 (−5.65 and −51.83 mA cm^−2^), and NiNx-800 (−0.87 and −44.38 mA cm^−2^). The higher current density of NiNx-600 with less Ni loading than NiNx-500 and Ni NPs suggest the important role of atomically dispersed Ni catalysts in promoting ECO_2_R. Although XC-72R and Ni NPs are able to achieve a high total current density, they have a very low CO selectivity with a maximum FE_CO_ of about 20% at the potential range of −0.6 V to −1.2 V vs. RHE (Figure 2B) due to the lack of effective active sites for selective CO_2_RR over HER. Moreover, NiNx-600 demonstrates a higher selectivity than other samples over the entire potential range, with a maximum FE_CO_ of 99%, which is among the best electrocatalysts for ECO_2_R to CO (Supplementary Table S3). Figure 2D shows the potential-determined CO partial current density (j_CO_). NiNx-600 achieves a j_CO_ of 5.89 mA cm^−2^ at −0.6 V vs. RHE, which is 512- and 78-fold higher than that of XC-72R and Ni NPs, respectively, verifying that the catalytic activity of NiNx-600 originates from plenty of atomically dispersed and pyrrolic N-coordinated Ni species.

**FIGURE 3:**
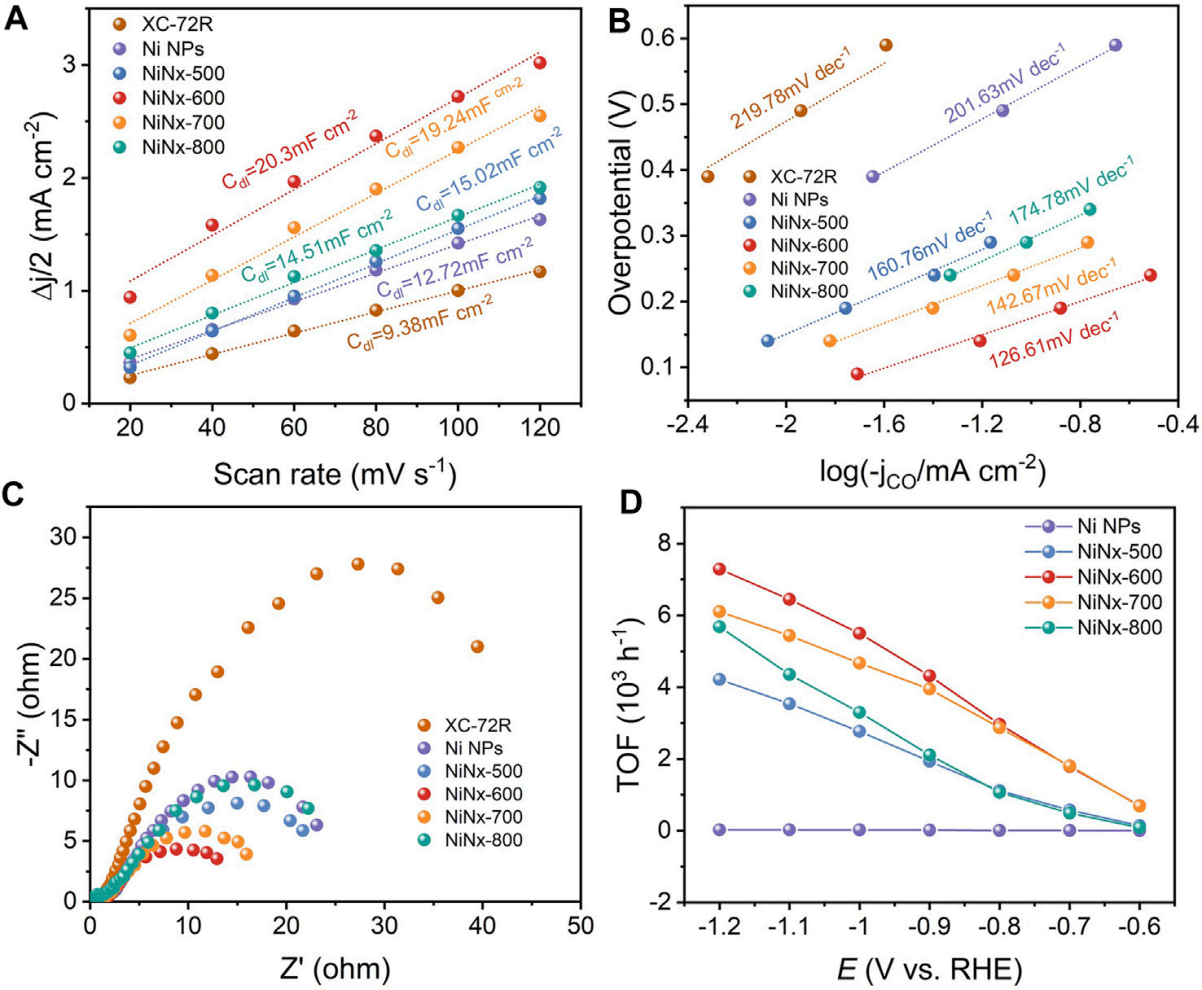
**(A)** Double layer capacitances. **(B)** Tafel plots and **(C)** Nyquist plots of XC-72R, Ni NPs, NiNx-500, NiNx-600, NiNx-700, and NiNx-800, respectively. **(D)** TOFs at different applied potentials.

**FIGURE 4:**
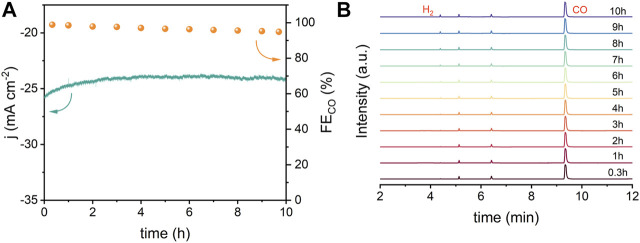
**(A)** Long-term stability of NiNx-600 at −0.8 V vs. RHE and **(B)** corresponding gas chromatography spectra.

The electrochemical active surface (ECSA) was evaluated from double-layer capacitance (C_dl_) to clarify the source of the excellent catalytic performance of Ni–N sites. As shown in Figure 3A and Supplementary Figure S18, NiNx-600 achieves a substantial C_dl_ of 20.3 mF cm^−2^ and thus a higher ECSA than other NiNx-T samples, Ni particles and XC-72R carbon, which is beneficial to expose more active sites for enhancing the ECO_2_R performance (Duan et al., 2018). Tafel plots were carried out to reveal the reaction kinetics on different active sites (Figure 3B). Tafel slopes of 219.78, 201.63, 160.76, 174.78,142.67, and 126.61 mV dec^−1^ were fitted for XC-72R, Ni NPs, NiNx-500, NiNx-800, NiNx-700, and NiNx-600, respectively. These results reveal that the rate-determining step of ECO_2_R on Ni–N sites for NiNx-600 is the proton-coupled electron transfer step to form the *COOH intermediate (Kong et al., 2021) as its Tafel slope is close to 118 mV dec^−1^. Notably, the markedly lower Tafel slope of NiNx-600 indicates enhanced reaction kinetics in the ECO_2_R process compared to the other counterparts (Duan et al., 2021). The Nyquist plots were acquired from electrochemical impedance spectroscopy (EIS) measurements. NiNx-T samples exhibit a lower charge transfer resistance compared to the bare XR-72R carbon carrier and Ni NPs, implying the highly dispersed Ni species on the carbon carriers favor a faster electron transfer kinetics than bulk Ni particles (Figure 3C). Among NiNx-T samples, NiNx-600 shows the lowest impedance, which originates from the promoted electron transfer kinetics by the higher content of atomic dispersed Ni species and the uniformly dispersed nickel species on the carbon carrier (Figure 1B and Supplementary Figures S9–S11). These all together endow NiNx-600 with an outperforming ECO_2_R activity than other contrast catalysts. The intrinsic activity of Ni-containing catalysts was uncovered by calculating the turnover frequency (TOF). The calculated TOFs at −1.2 V vs. RHE for Ni NPs and NiNx-Ts are 26, 4,217, 7,291, 6,106, and 5,686 h^−1^, respectively. The highest TOF for NiNx-600 suggests the atomic Ni–N sites, and the dominance of single-atom combined with cluster sites greatly contribute to the highly boosted activity in ECO_2_R (Figure 3D). The stability of NiNx-600 during long-term electrocatalysis is shown in Figure 4. The total current density exhibits a negligible attenuation and its FE_CO_ maintains 96% of the initial FE, manifesting the good stability of Ni–N sites in NiNx-600 in electrocatalytic ECO_2_R. The comparison of XRD patterns, Raman spectra, and high-resolution Ni 2p XPS spectra for NiNx-600 before and after 10 h electrolysis also manifests the structure and chemical state of the carbon carrier and Ni species (Supplementary Figures S20A–C). The HAADF-STEM image in Supplementary Figure S20 indicates the absence of the formation of bulk Ni species during the catalytic process and the maintenance of the atomic-level dispersion. During the ECO_2_R process, the valence state of the Ni active center decreases because electrons in the catalyst are transferred to the reactants and intermediates. The performance comparison of NiNx-600 and other single-atom catalysts further highlights the superiority of the ensemble of single atoms and clusters in boosting the high-efficiency ECO_2_R process (Supplementary Table S3).

## Conclusion

In summary, we have synthesized a series of atomically dispersed Ni single-atom and cluster catalysts for boosting ECO_2_R to CO by importing the pyrrolic N-coordinated Ni species on the carbon carrier. Upon modulation of the dispersion of Ni species and the content of pyrrolic N moieties through temperature control, NiNx-600 with well-dispersed Ni-pyrrolic N single-atom and cluster sites demonstrates a considerable electrocatalytic ECO_2_R performance with a maximum j_CO_ of 61.85 mA cm^−2^ and corresponding highest FE_CO_ of 99%, as well as excellent TOF of 7,291 h^−1^. The contrast results indicate that the integration of single atoms and clusters is greatly superior to single-atom catalysts in ECO_2_R, wherein pyrrolic N-coordinated Ni species play an important role in promoting the ECO_2_R process for NiNx-600. This work highlights the significance of regulating the coordination of transition metal sites and the synergistic effect between the isolated single-site and cluster site in enhancing the ECO_2_R performance, which can be expanded to other complicated electrocatalysis such as nitrogen reduction, oxygen evolution, and nitrate reduction reaction.

## Data Availability

The raw data supporting the conclusion of this article will be made available by the authors, without undue reservation.
