# Supplementary material for: Boosting Electrochemical Carbon Dioxide Reduction on Atomically Dispersed Nickel Catalyst
Source: Front Chem. 2022 Jan 20;9:837580. doi: 10.3389/fchem.2021.837580 (PMC8811444; doi:10.3389/fchem.2021.837580)
Supplement: Supplementary file 1 [file DataSheet1.docx]

Supplementary Material

**Boosting Electrochemical Carbon Dioxide Reduction on Atomically Dispersed Nickel Catalyst**

**Qi Hao^1, 3^, Dong-Xue Liu^1^, Ruiping Deng^3^, Hai-Xia Zhong^2, 3*^**

^1^Key Laboratory of Automobile Materials, Ministry of Education, School of Materials Science and Engineering, Jilin University, Changchun, China

^2^Center for Advancing Electronics Dresden (cfaed) and Faculty of Chemistry and Food Chemistry, Technische Universität Dresden, Dresden, Germany

^3^State Key Laboratory of Rare Earth Resource Utilization, Changchun Institute of Applied Chemistry, Chinese Academy of Sciences, Changchun, China

*** Correspondence:**Hai-Xia Zhong
haixia.zhong @tu-dresden.de

Supplementary Figures and Tables


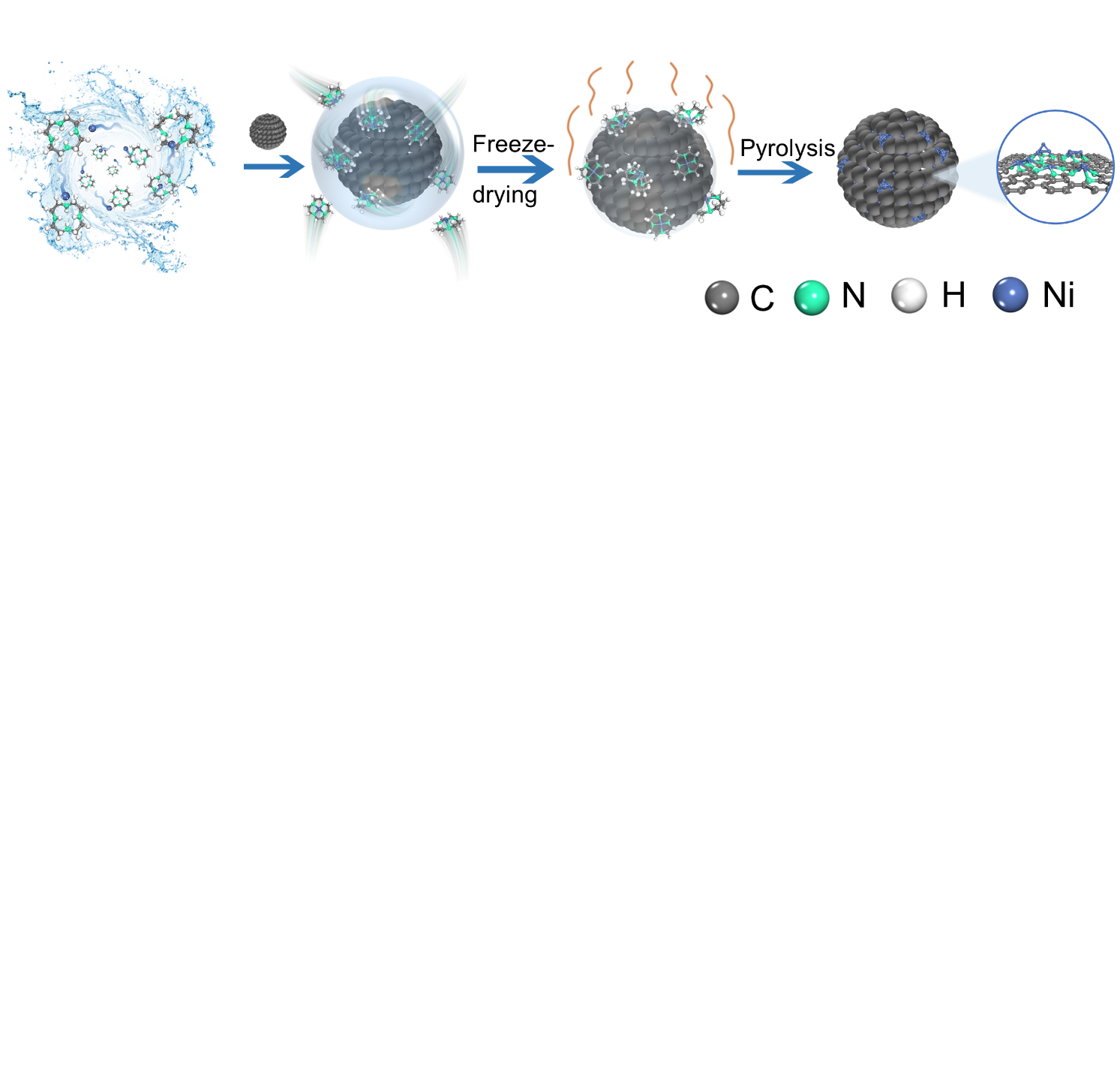


**Supplementary Figure 1.** Illustration of the synthesis procedure of NiNx-600.


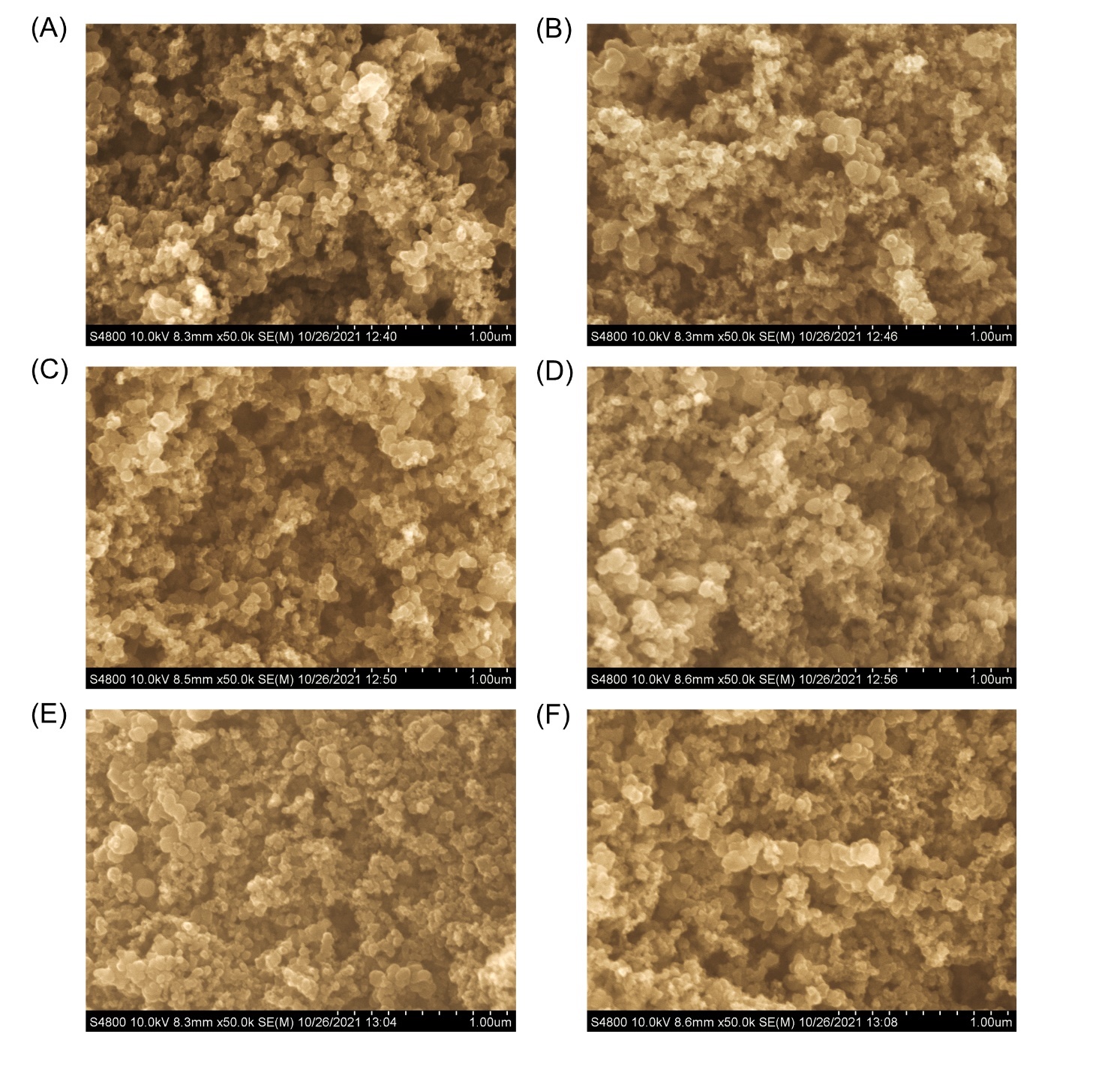


**Supplementary Figure 2.** (**A-F**) SEM images of XC-72R, Ni NPs, NiNx-500, NiNx-600, NiNx-700 and NiNx-800, respectively.


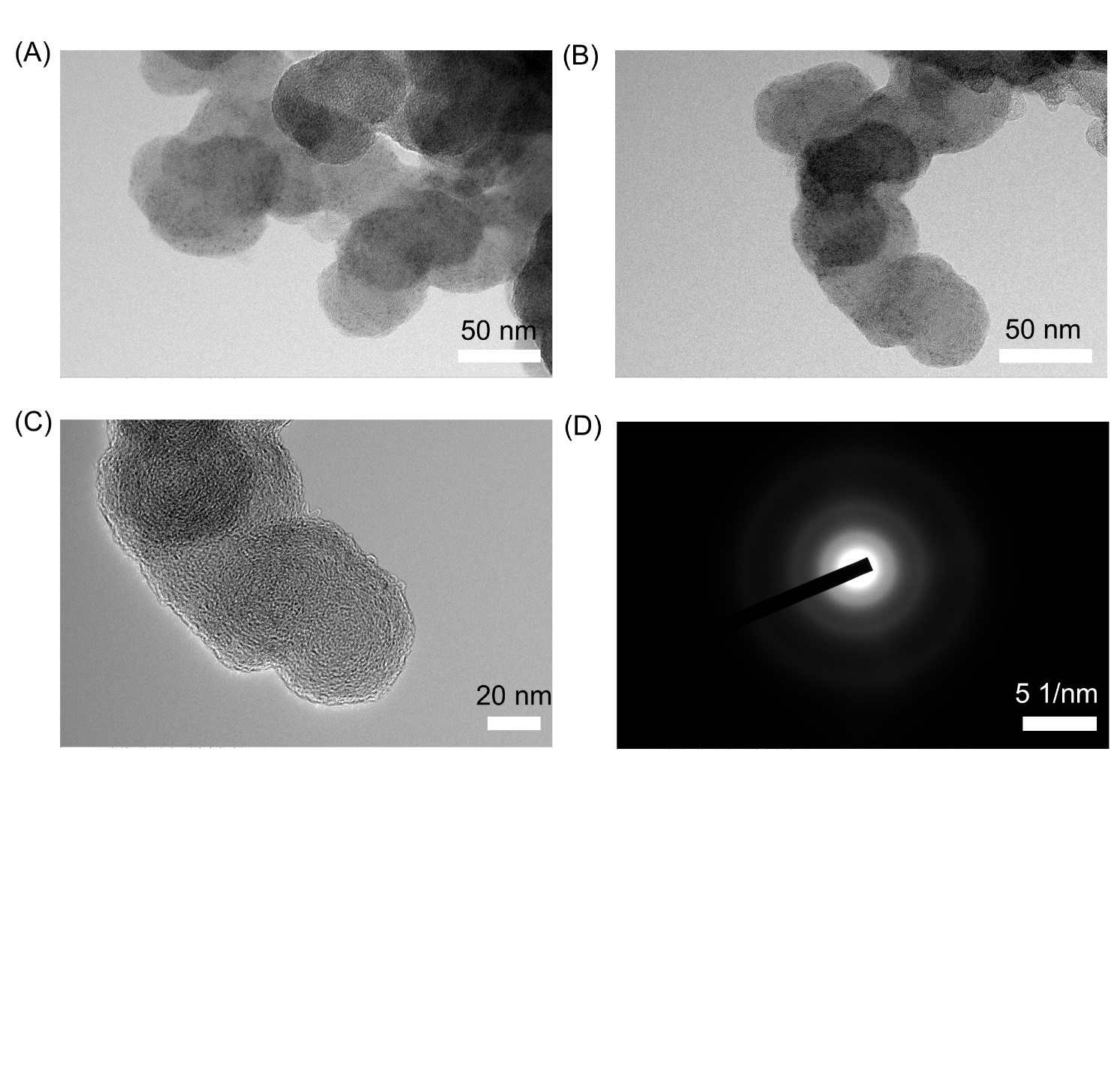


**Supplementary Figure 3.** (**A, B**) TEM images. (**C**) HRTEM image and (**D**) SAED patterns of XC-72R.


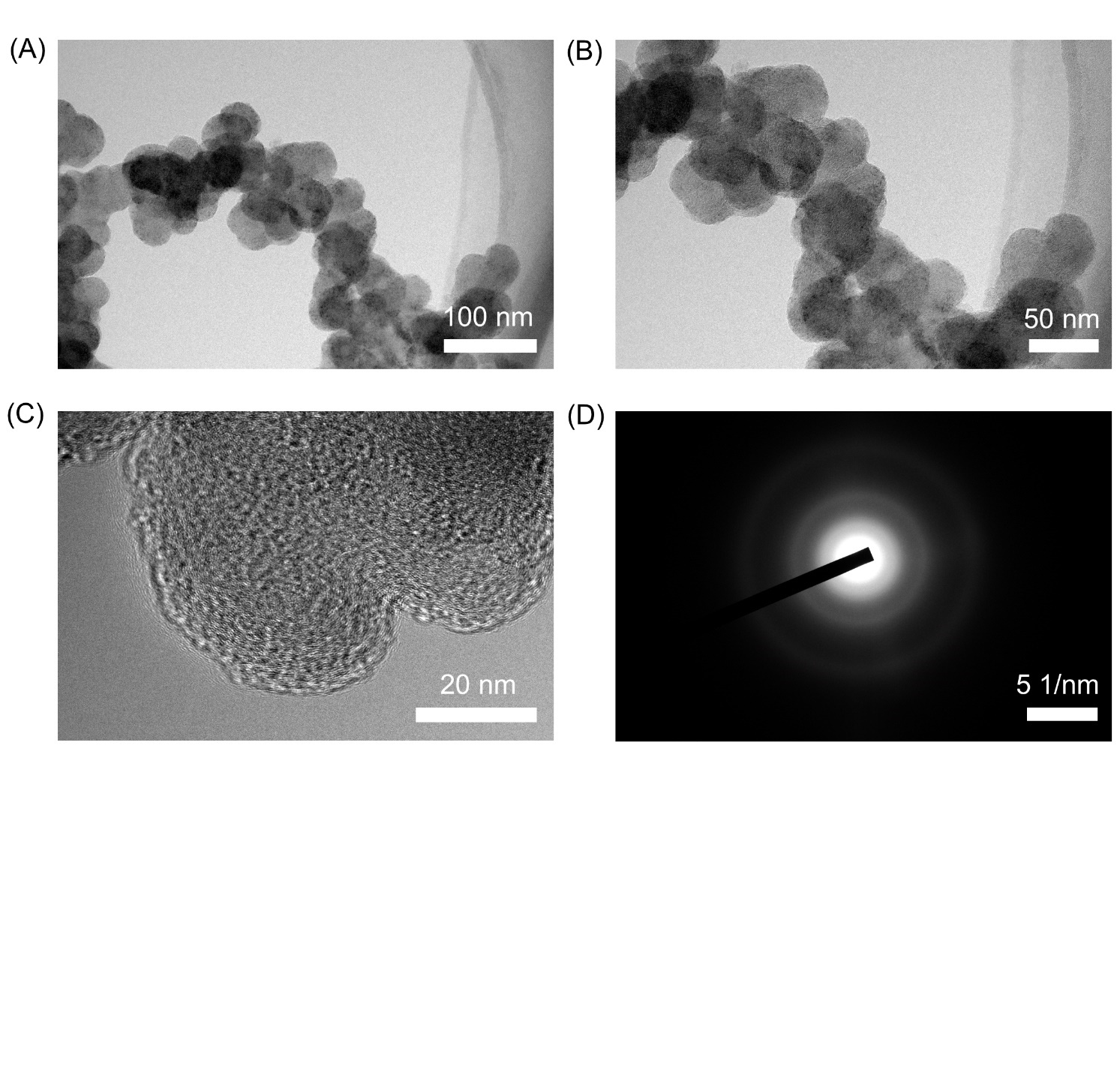


**Supplementary Figure 4.** (**A, B**) TEM images. (**C**) HRTEM image and (**D**) SAED patterns of NiNx-500.


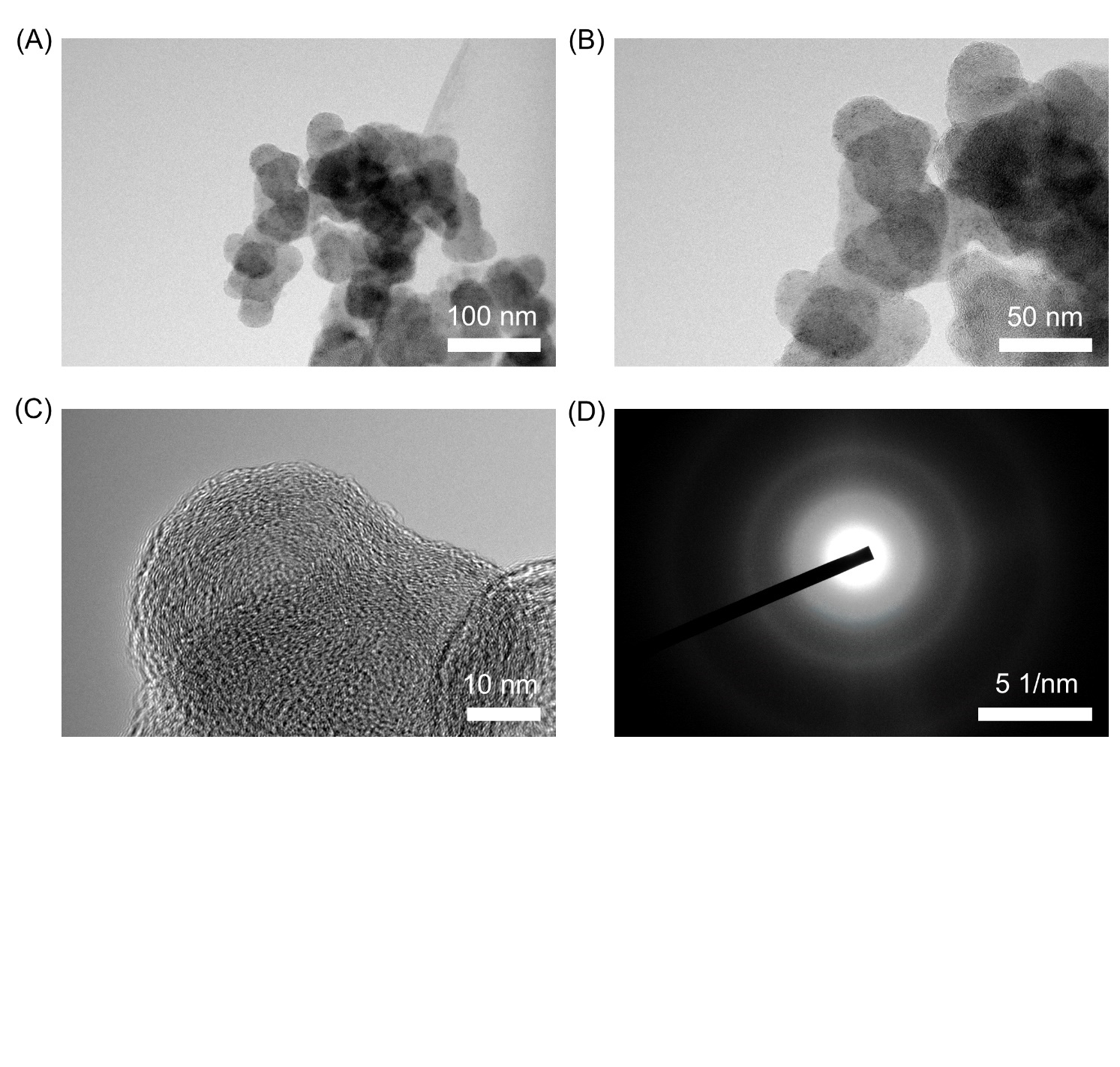


**Supplementary Figure 5.** (**A, B**) TEM images. (**C**) HRTEM image and (**D**) SAED patterns of NiNx-600.


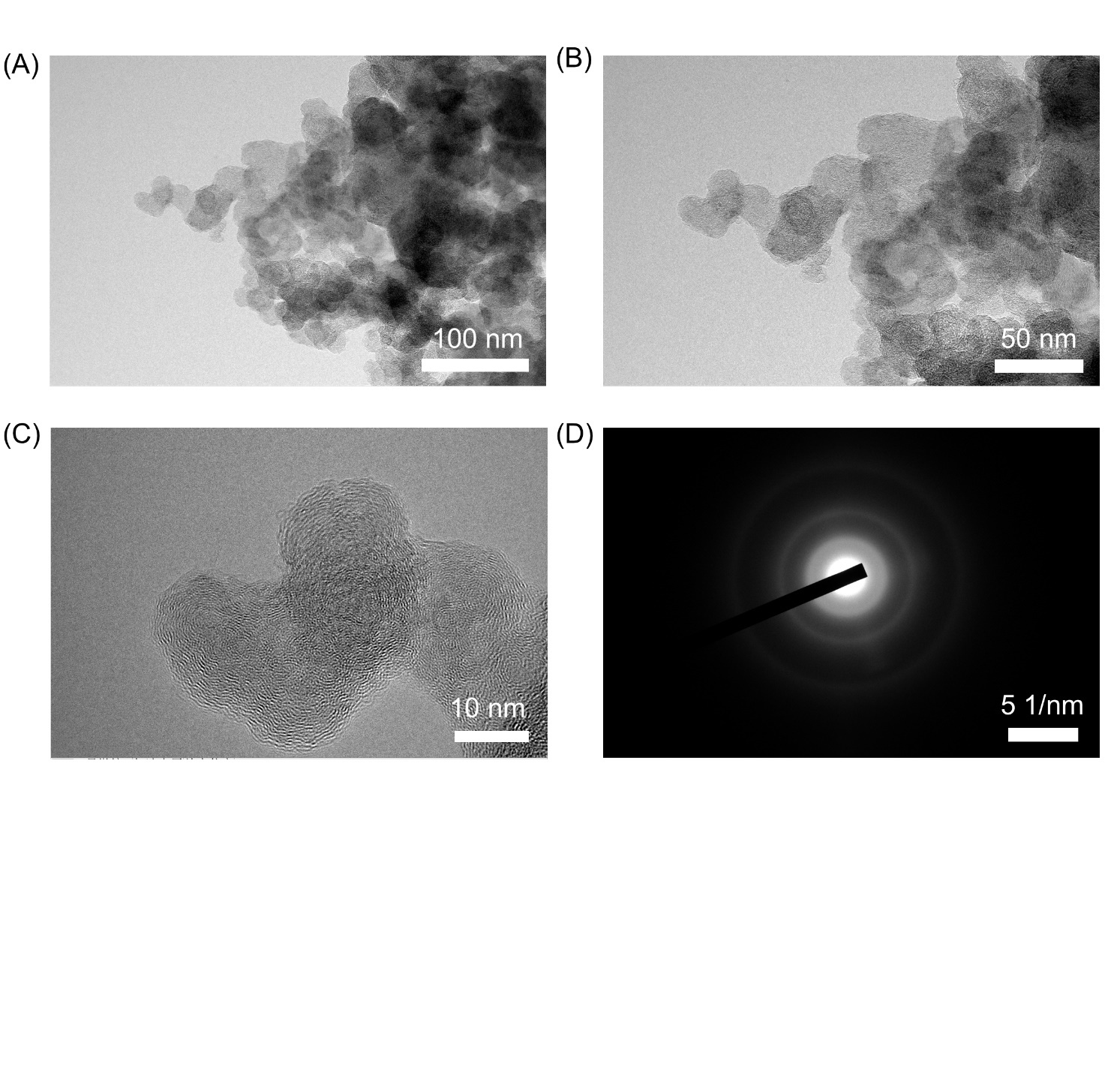


**Supplementary Figure 6.** (**A, B**) TEM images. (**C**) HRTEM image and (**D**) SAED patterns of NiNx-700.


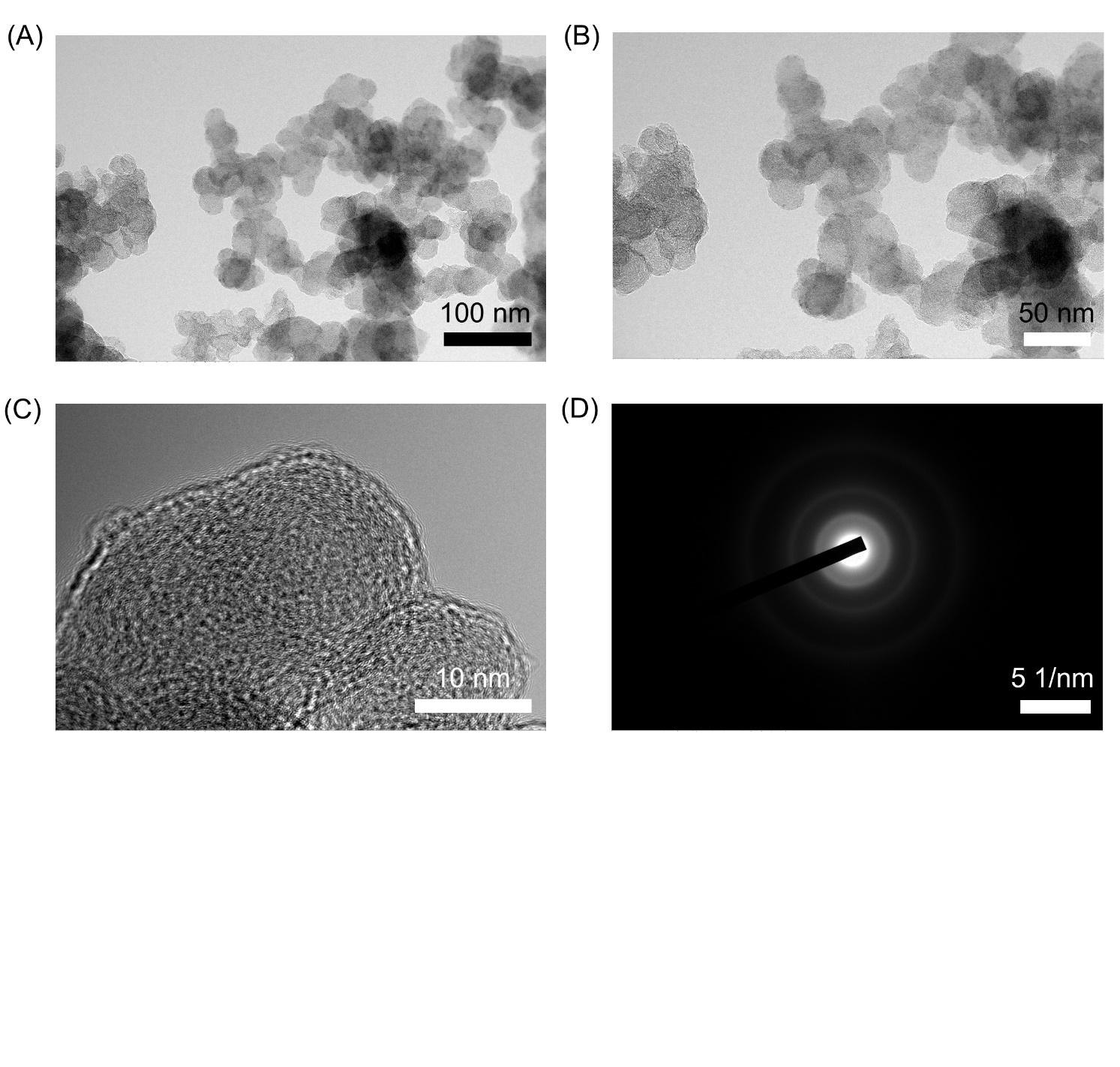


**Supplementary Figure 7.** (**A, B**) TEM images. (**C**) HRTEM image and (**D**) SAED pattern of NiNx-800.


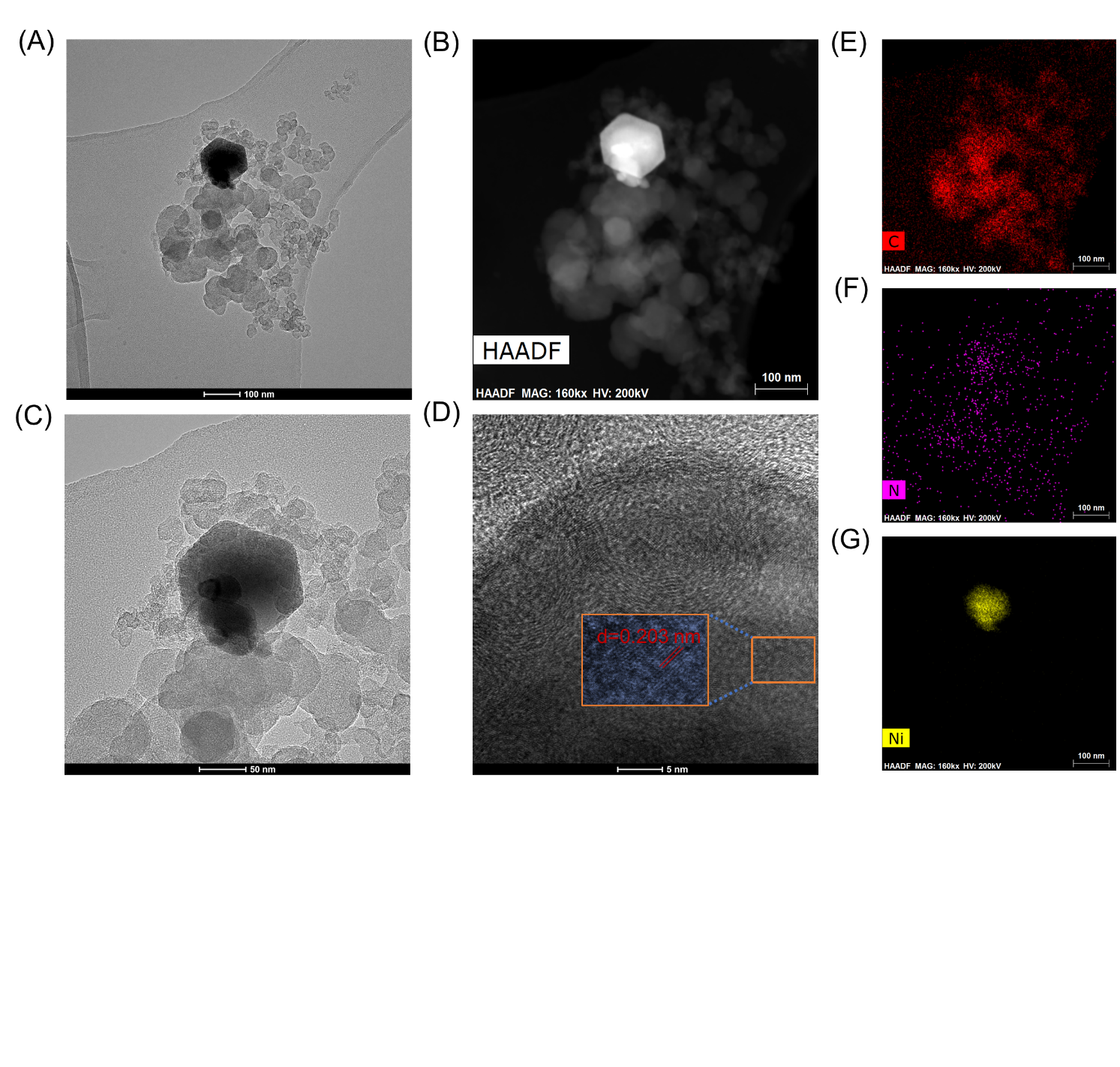


**Supplementary Figure 8.** (**A-C**) TEM images. (**D**) HRTEM images. (**E-F**) Corresponding EDX elemental mapping of Ni NPs.


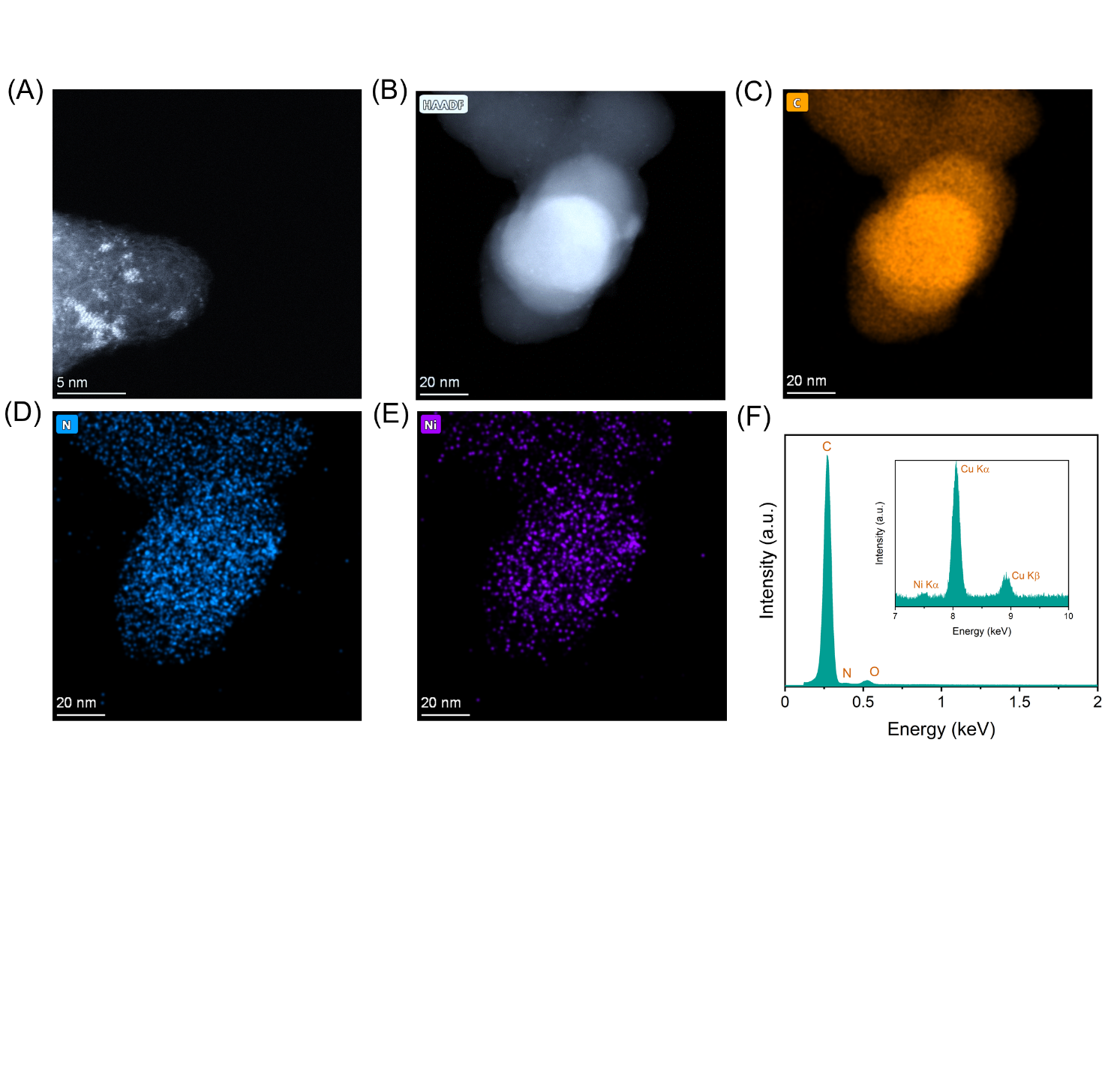


**Supplementary Figure 9.** (**A, B**) HAADF-STEM images. (**C-E**) Corresponding EDX elemental mapping of C, N and Ni element, respectively, and (**F**) EDX analysis of NiNx-500.


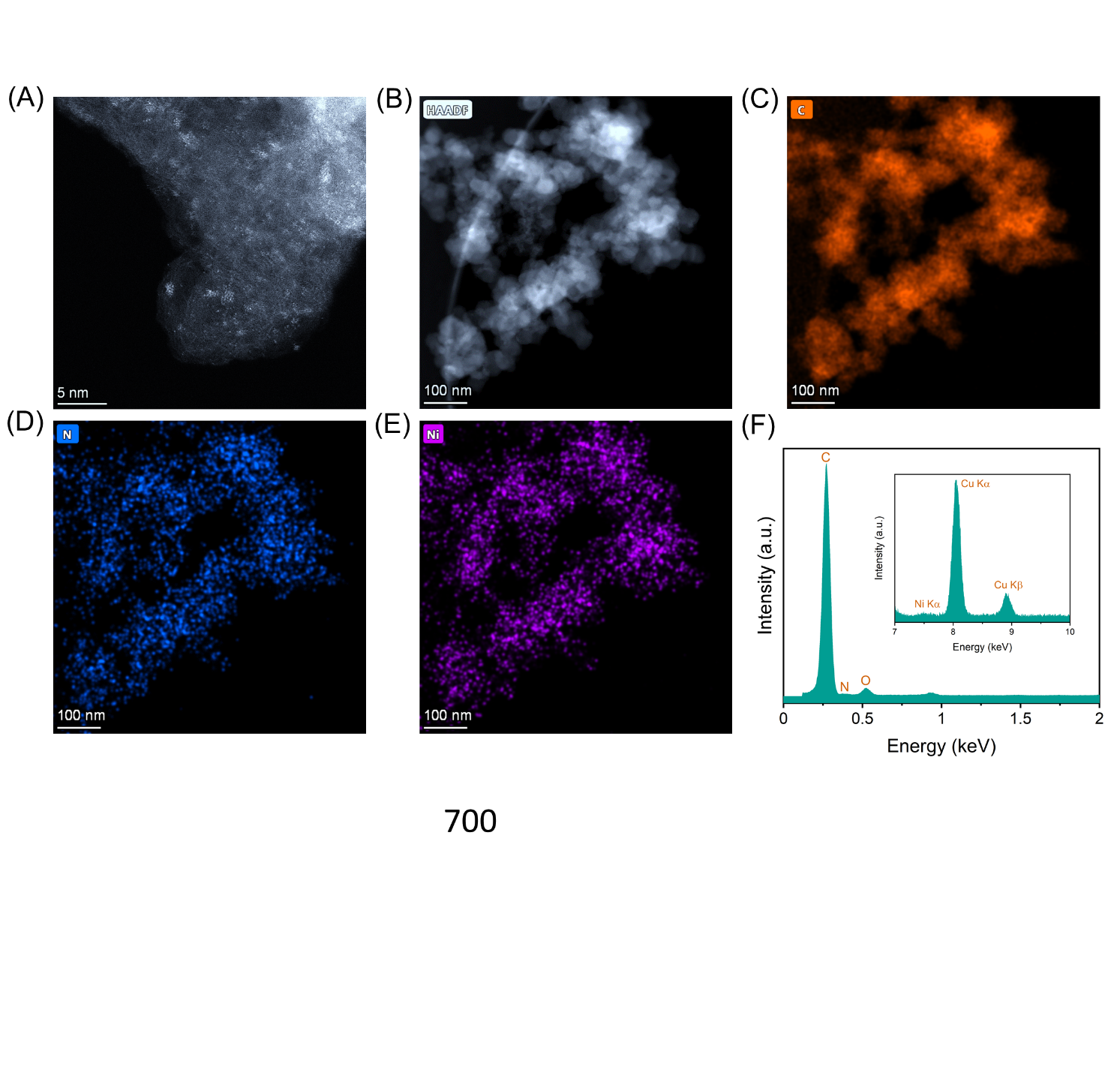


**Supplementary Figure 10.** (**A, B**) HAADF-STEM images. (**C-E**) Corresponding EDX elemental mapping of C, N and Ni element, respectively, and (**F**) EDX analysis of NiNx-700.


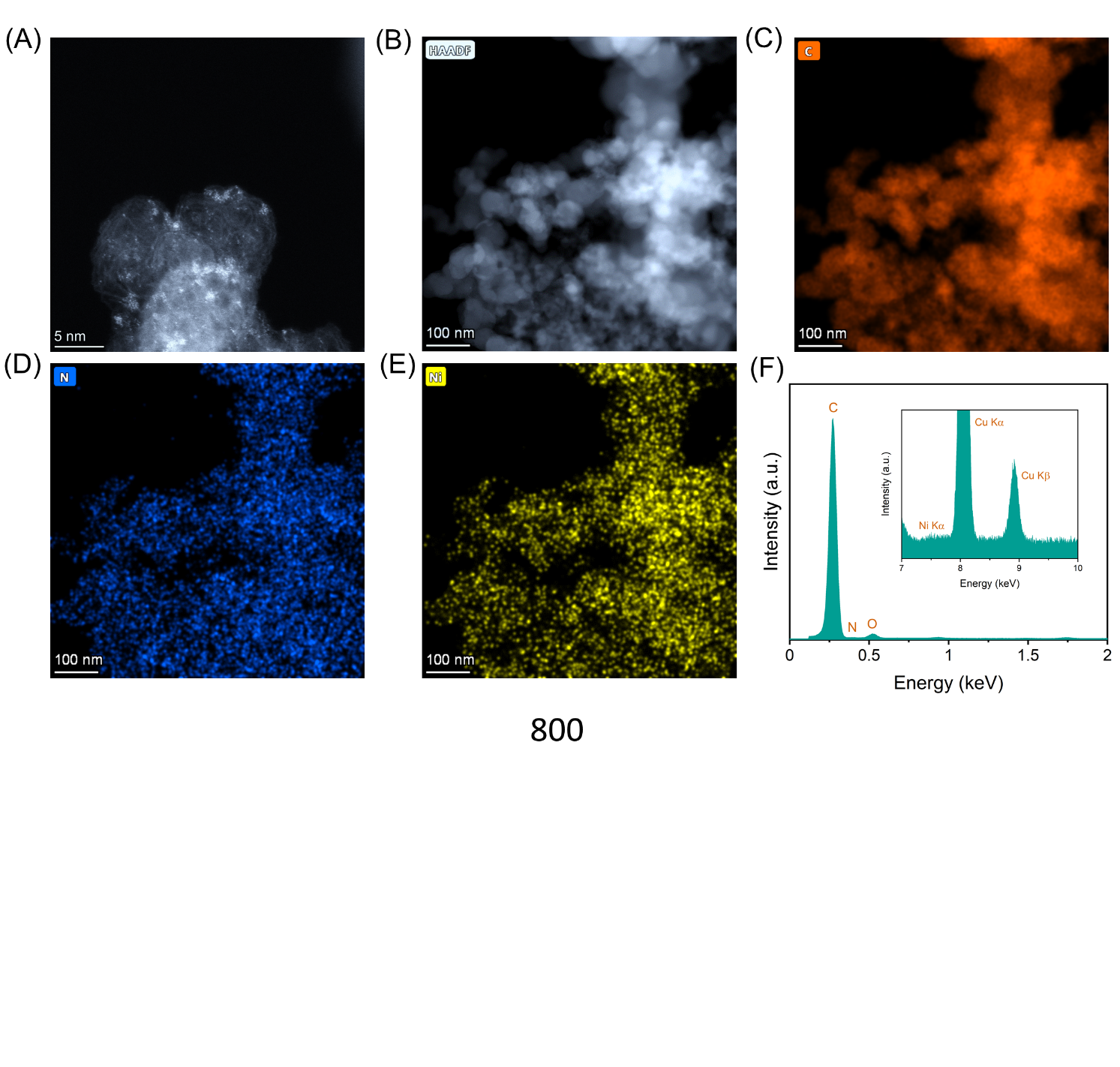


**Supplementary Figure 11.** (**A, B**) HAADF-STEM images. (**C-E**) Corresponding EDX elemental mapping of C, N and Ni element, respectively, and (**F**) EDX analysis of NiNx-800.


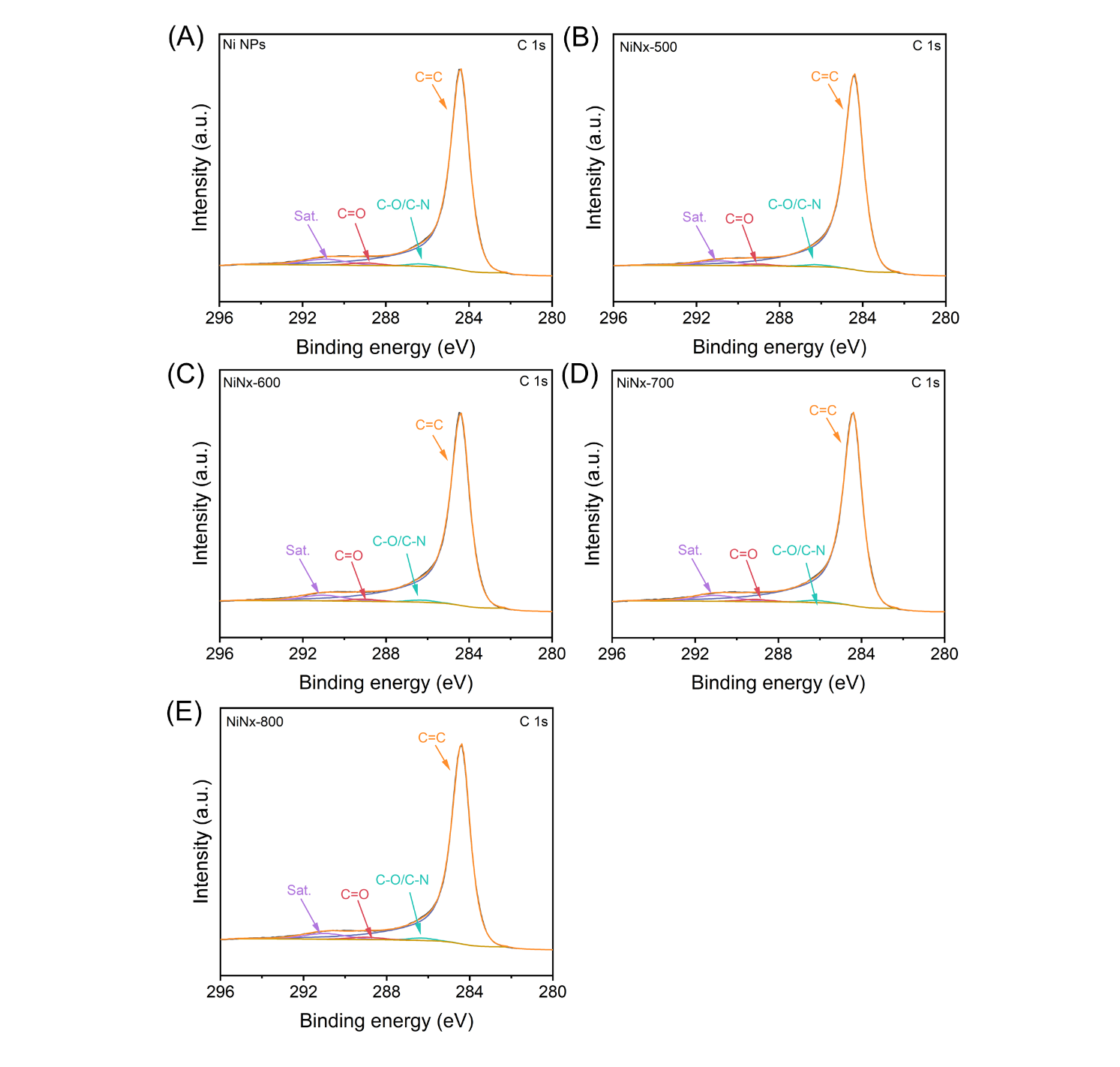


**Supplementary Figure 12.** (**A-E**) High-resolution C 1s XPS spectra of Ni NPs, NiNx-500, NiNx-600, NiNx-700 and NiNx-800, respectively.


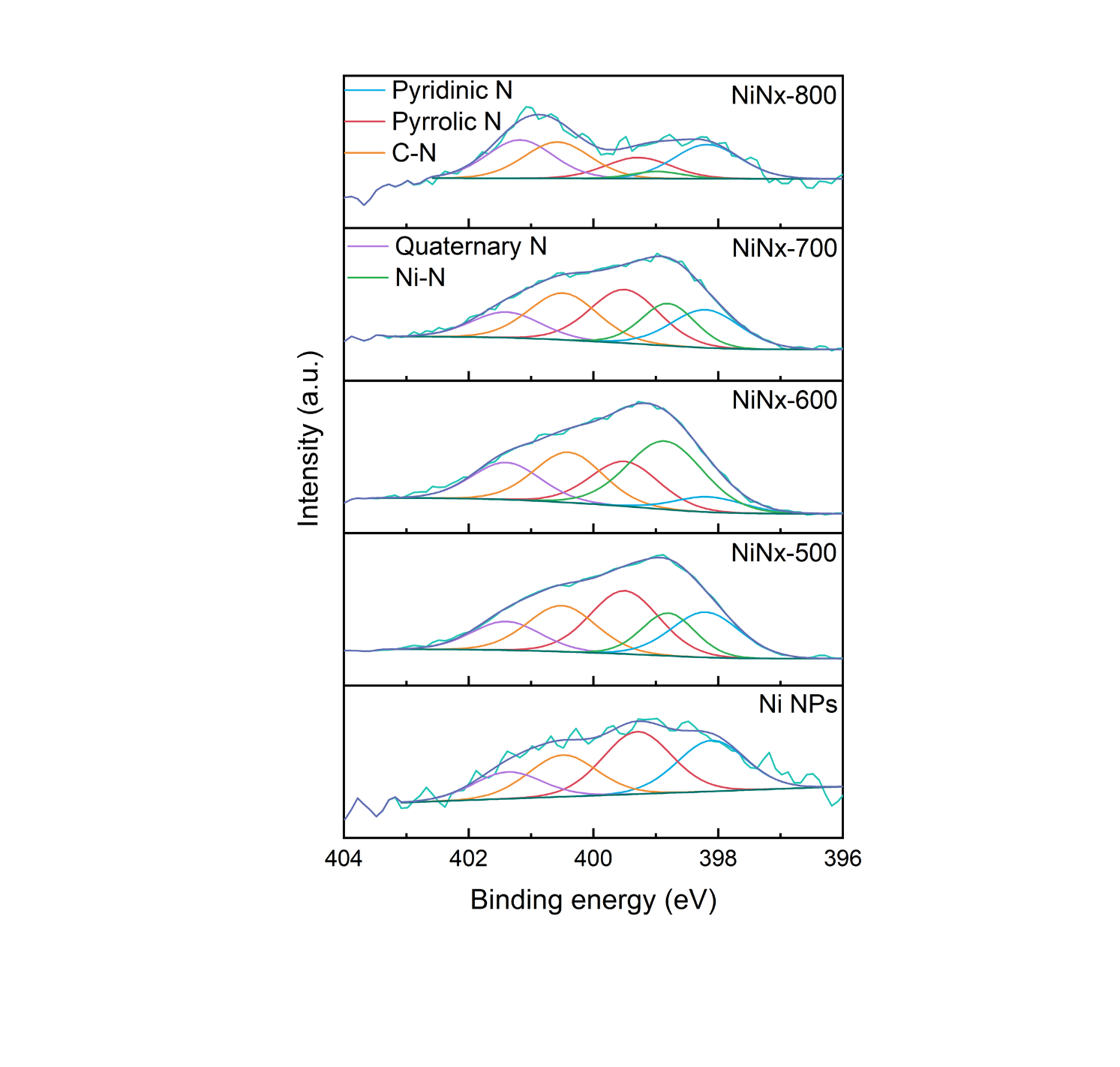


**Supplementary Figure 13.** High-resolution N 1s spectra of Ni NPs, NiNx-500, NiNx-600, NiNx-700 and NiNx-800, respectively.

The content of pyrrolic N and pyridinic N is 0.32 and 0.11 at% respectively. It is obvious that the content of pyrrolic N is almost three times higher than that of pyridinic N, which suggests that most nickel atoms are coordinated with pyrrolic N instead of pyridinic N.


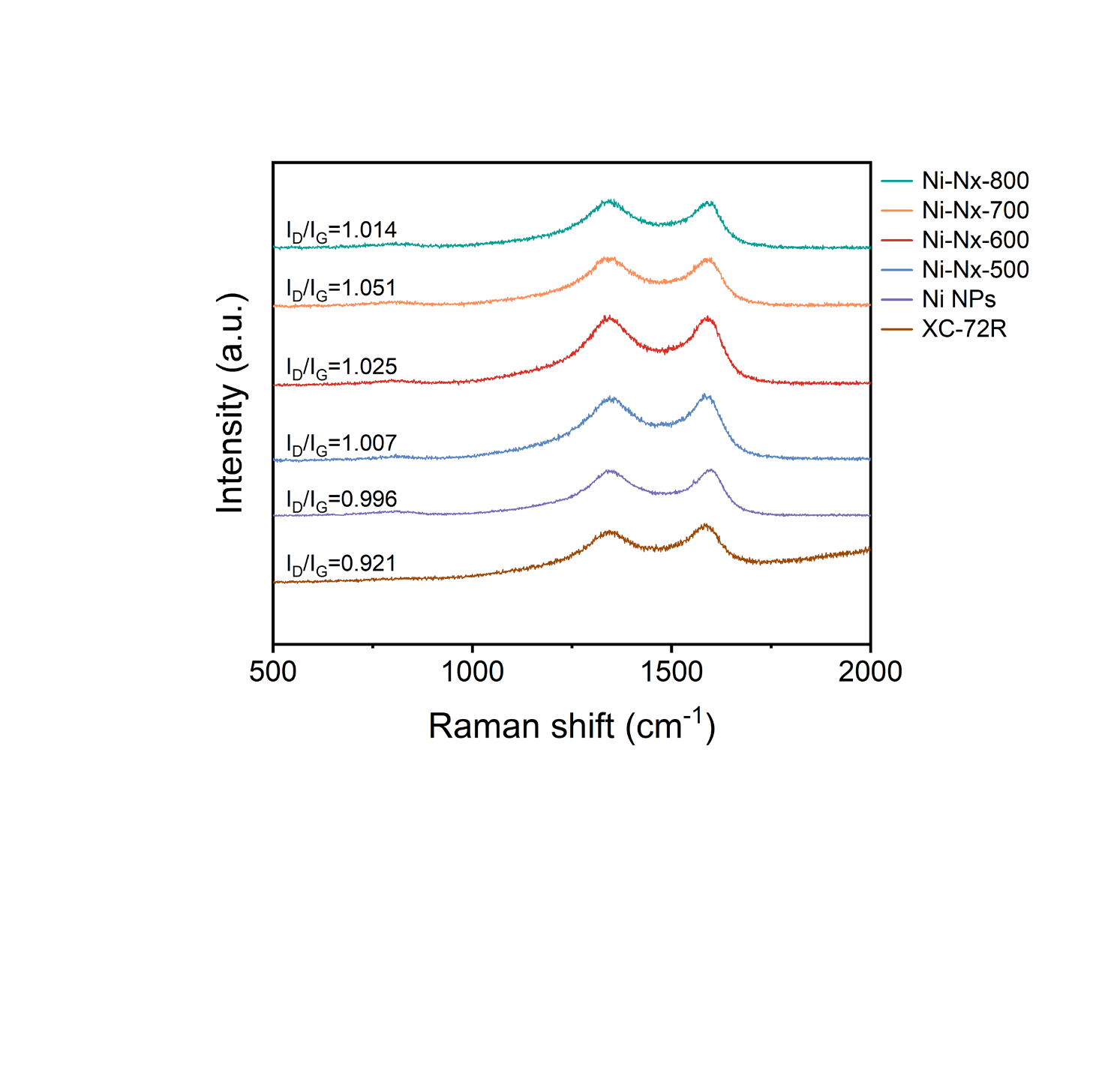


**Supplementary Figure 14.** Raman spectra of XC-72R, Ni NPs, NiNx-500, NiNx-600, NiNx-700 and NiNx-800, respectively.


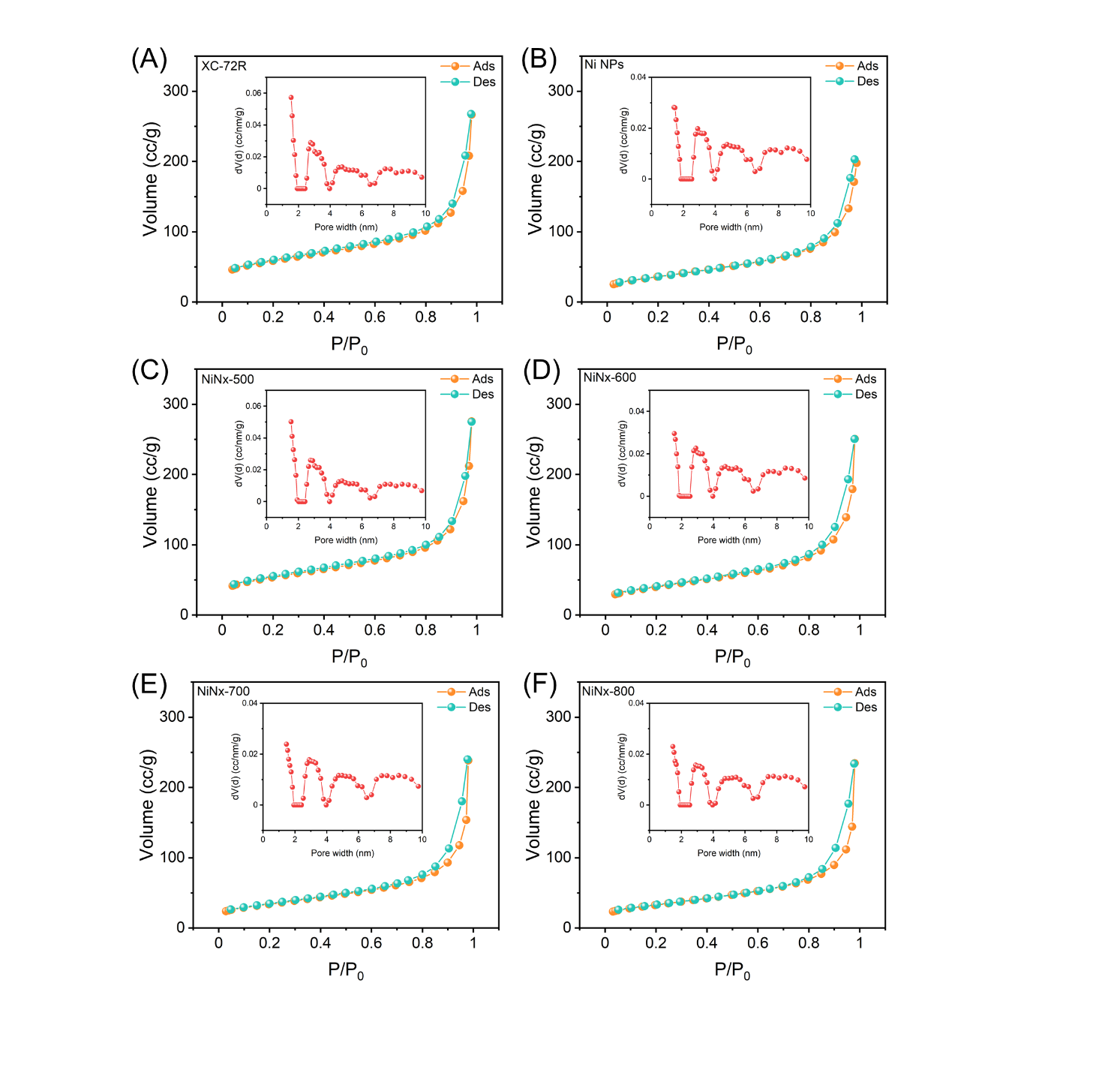


**Supplementary Figure 15.** (**A-F**) Nitrogen adsorption and desorption isotherms at 77.3K and corresponding pore size distributions of XC-72R, Ni NPs, NiNx-500, NiNx-600, NiNx-700 and NiNx-800, respectively.


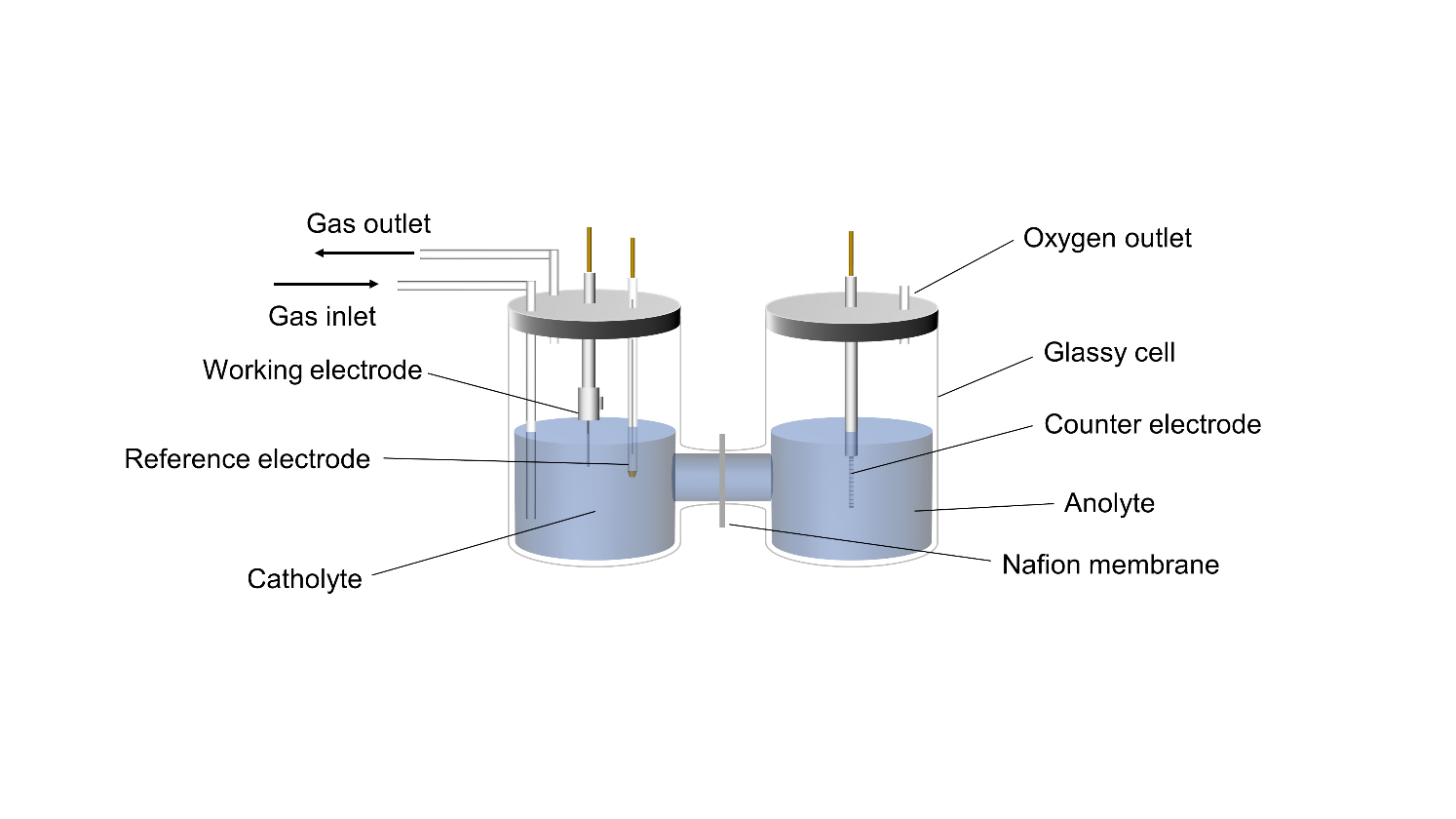


**Supplementary Figure 16.** Illustration of the three-electrode H-type cell for ECO_2_R.

Carbon paper loaded with catalyst was serviced as the working electrode while Pt and Ag/AgCl were separately used as counter and reference electrode. Nafion 212 film was used separated membrane in this H-type electrochemical cell. 0.5M KHCO_3_ solution is the electrolyte.


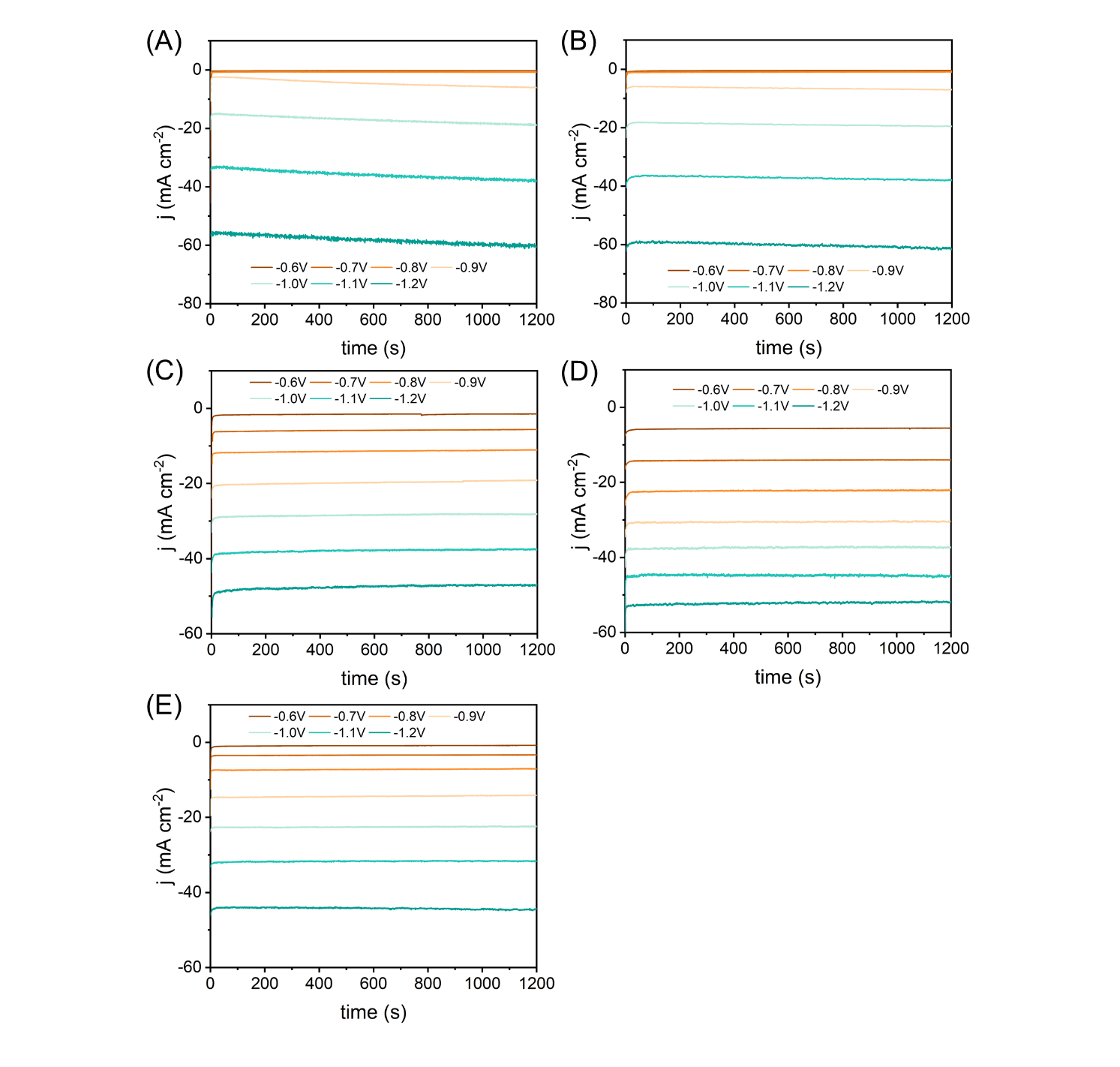


**Supplementary Figure 17.** (**A-E**) CA curves at different applied potentials of XC-72R, Ni NPs, NiNx-500, NiNx-700 and NiNx-800, respectively.


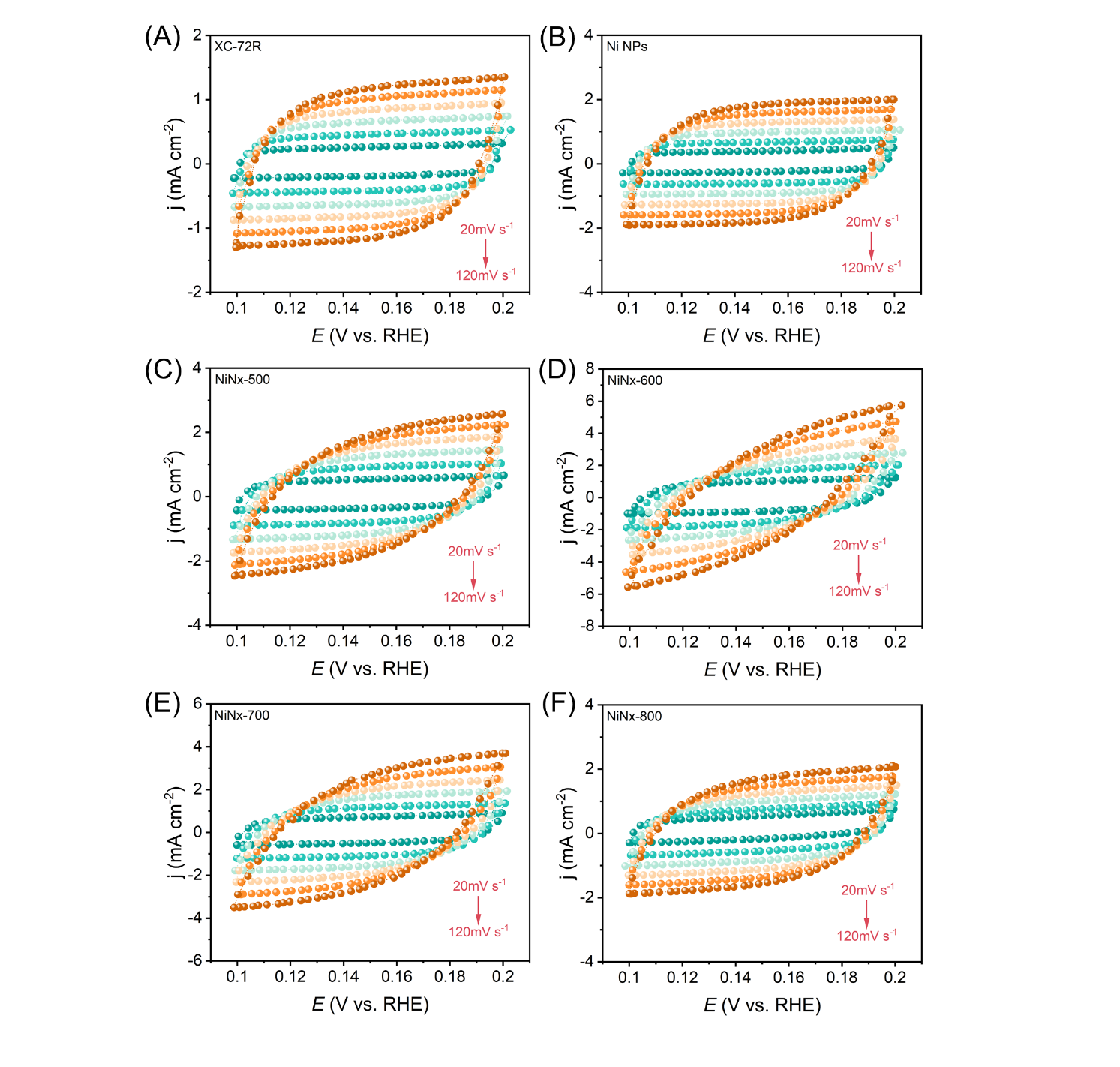


**Supplementary Figure 18.** (**A-F**) CV measurements with scan ranging from 20 to 120 mV s^-1^ with an interval of 20 mV s^-1^ at the potential range of 0.1 V to 0.2 V vs. RHE for XC-72R, Ni NPs, NiNx-500, NiNx-600, NiNx-700 and NiNx-800, respectively.


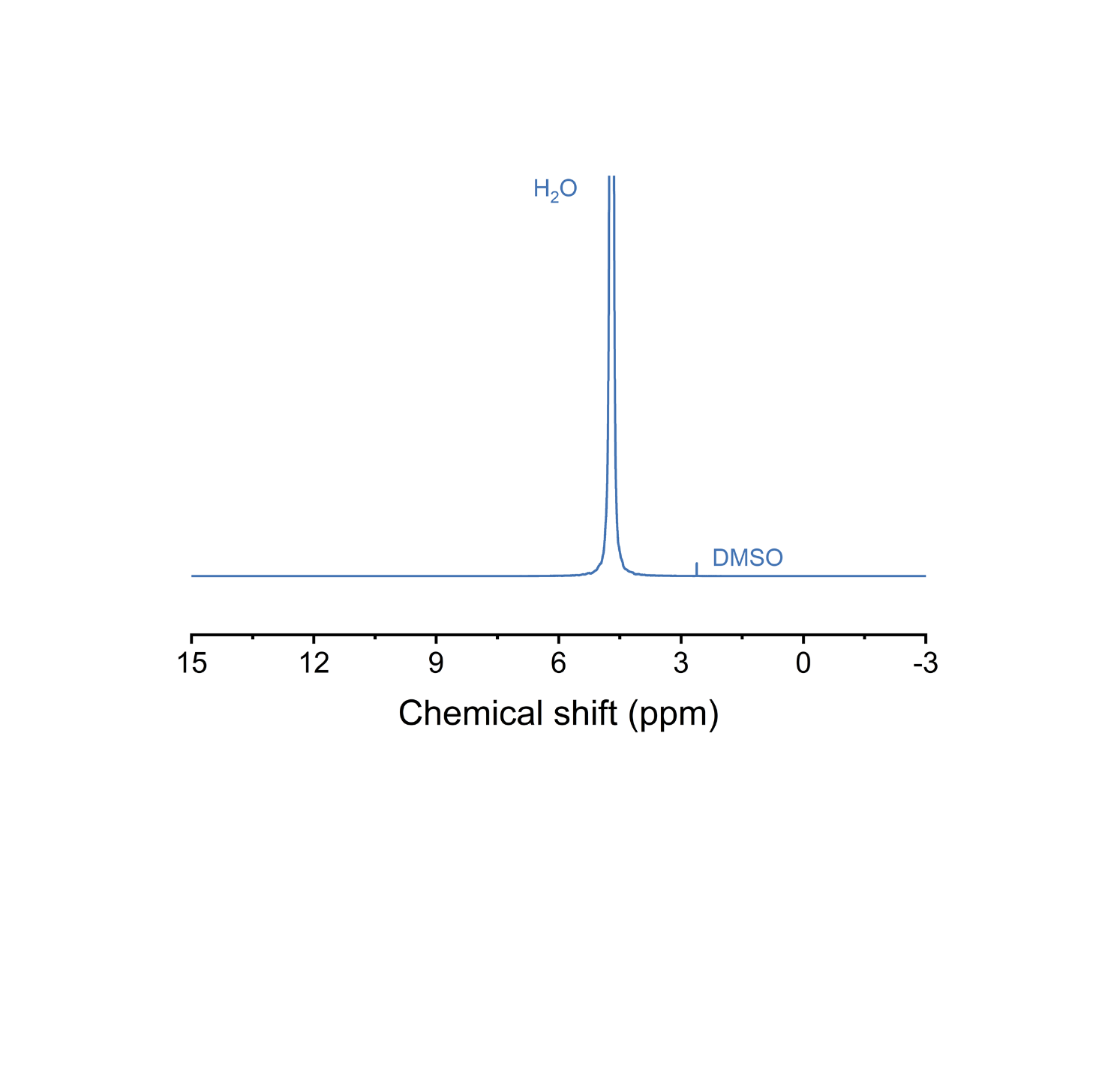


**Supplementary Figure 19.** Detection for the liquid product of NiNx-600 after 1 h ECO_2_R at -0.8 V vs. RHE.


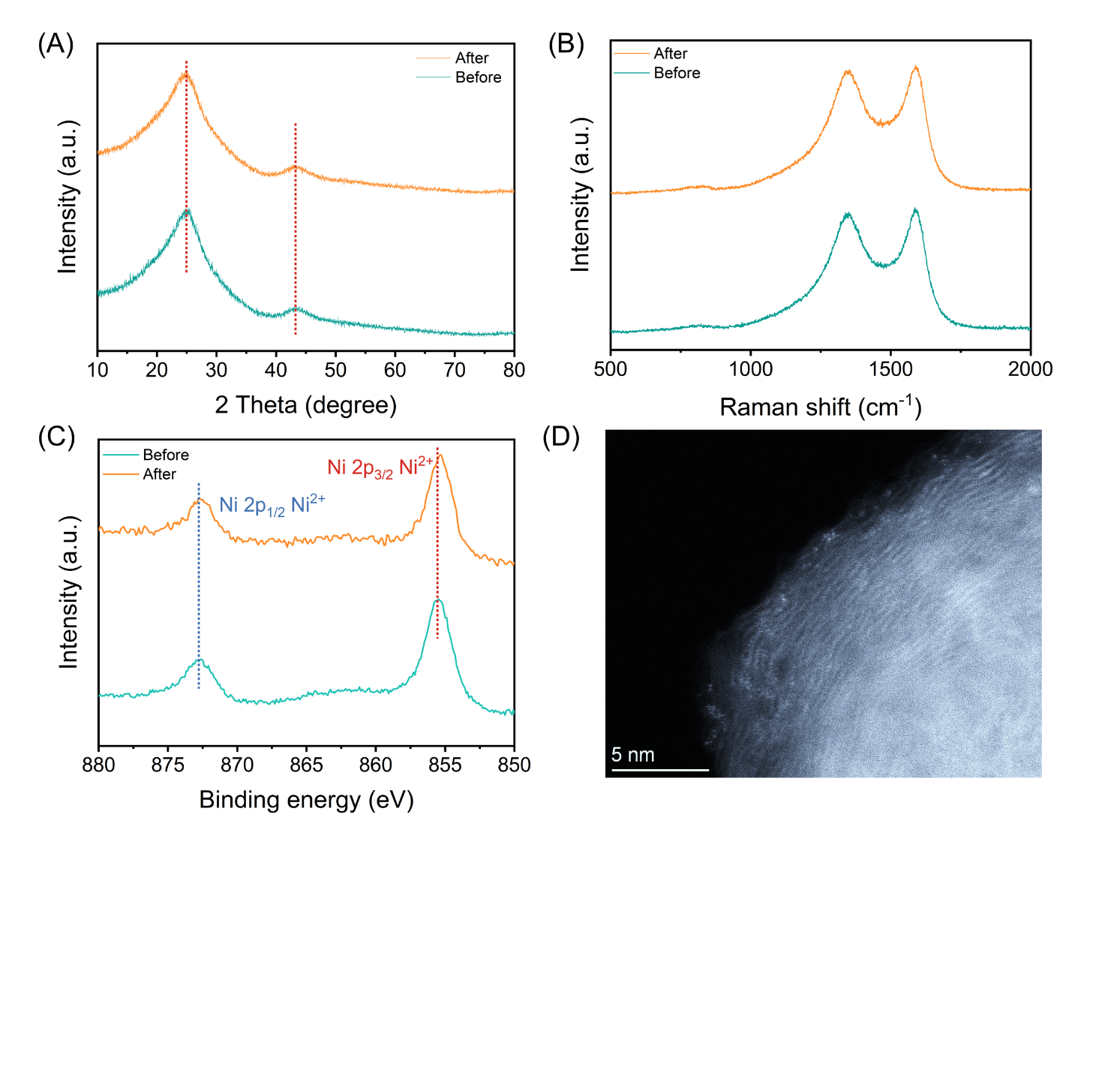


**Supplementary Figure 20.** (**A-C**) Comparison of XRD patterns (**A**), Raman spectra (**B**) and high-resolution Ni 2p XPS spectra (**C**) for NiNx-600 before and after 10 h electrocatalysis. (**D**) HAADF-STEM image of NiNx-600 after 10 h electrocatalysis.

**Supplementary Table 1.** The BET surface area of different samples.

| Sample | BET surface area (m^2^ g^-1^) |
| --- | --- |
| XC-72R | 208.9 |
| Ni NPs | 120.7 |
| NiNx-500 | 190.0 |
| NiNx-600 | 142.0 |
| NiNx-700 | 128.4 |
| NiNx-800 | 116.8 |

**Supplementary Table 2.** The Ni content of different samples based on ICP-OES.

| Sample | Ni wt.% |
| --- | --- |
| Ni NPs | 1.77 |
| NiNx-500 | 0.60 |
| NiNx-600 | 0.52 |
| NiNx-700 | 0.46 |
| NiNx-800 | 0.13 |

**Supplementary Table 3.** Comparation of carbon-based Ni SACs with our Ni clusters catalyst toward ECO_2_R to CO

| Catalyst | FE_CO_ (%)  (Overpotential/V) | j_CO_ (mA cm^-2^) | Electrolyte | Ref. |
| --- | --- | --- | --- | --- |
| **NiNx-600** | **93.8 (1.09)** | **61.85(1.09)** | **0.5M KHCO_3_** | **This work** |
| **NiNx-600** | **97.1 (0.99)** | **54.67 (1.09)** | **0.5M KHCO_3_** | **This work** |
| **NiNx-600** | **98.7 (0.89)** | **46.6 (0.89)** | **0.5M KHCO_3_** | **This work** |
| **NiNx-600** | **99 (0.79)** | **36.6 (0.79)** | **0.5M KHCO_3_** | **This work** |
| Ni-N_3_-C | 95.6 (0.54) | ~18 (0.89) | 0.5M KHCO_3_ | (Zhang et al., 2021) |
| Ni-SAs@FNC | 97 (0.68) | 22 (0.68) | 0.5M KHCO_3_ | (Han et al., 2021) |
| NiNG | 92.7 (0.69) | 19.4 (0.79) | 0.5M KHCO_3_ | (Jia et al., 2021) |
| Ni/NCTs | 98 (0.89) | 34.3 (0.89) | 0.5M KHCO_3_ | (Hou et al., 2020) |
| Ni/HMCS-3-800 | ~95 (0.79) | 10.5 (0.89) | 0.5M KHCO_3_ | (Xiong et al., 2020) |
| Ni SAs/N-C | 71.9 (0.89) | 10.48 | 0.5M KHCO_3_ | (Zhao et al., 2017) |

Reference

Zhang, Y., Jiao, L., Yang, W., Xie, C. and Jiang, H.-L. (2021). Rational Fabrication of Low-Coordinate Single-Atom Ni Electrocatalysts by MOFs for Highly Selective CO_2_ Reduction. *Angew. Chem. Int. Ed.* 14, 7607-7611. doi: 10.1002/anie.202016219

Han, S.-G., Ma, D.-D., Zhou, S.-H., Zhang, K., Wei, W.-B., Du, Y., et al. (2021). Fluorine-tuned single-atom catalysts with dense surface Ni-N_4_ sites on ultrathin carbon nanosheets for efficient CO_2_ electroreduction. *Appl. Catal. B: Environ.* 283, 119591. doi: 10.1016/j.apcatb.2020.119591

Jia, C., Tan, X., Zhao, Y., Ren, W., Li, Y., Su, Z., et al. (2021). Sulfur-Dopant-Promoted Electroreduction of CO_2_ over Coordinatively Unsaturated Ni-N_2_ Moieties. *Angew. Chem. Int. Ed*. 43, 23342-23348. doi: 10.1002/anie.202109373

Hou, Y., Liang, Y.-L., Shi, P.-C., Huang, Y.-B. and Cao, R. (2020). Atomically dispersed Ni species on N-doped carbon nanotubes for electroreduction of CO_2_ with nearly 100% CO selectivity. *Appl. Catal. B: Environ.* 271, 118929. doi: 10.1016/j.apcatb.2020.118929

Xiong, W., Li, H., Wang, H., Yi, J., You, H., Zhang, S., et al. (2020). (2020). Hollow Mesoporous Carbon Sphere Loaded Ni–N_4_ Single-Atom: Support Structure Study for CO_2_ Electrocatalytic Reduction Catalyst. *Small* 41, 2003943. doi: 10.1002/smll.202003943

Zhao, C., Dai, X., Yao, T., Chen, W., Wang, X., Wang, J., et al. (2017). Ionic Exchange of Metal–Organic Frameworks to Access Single Nickel Sites for Efficient Electroreduction of CO_2_. *J. Am. Chem. Soc.* 24, 8078-8081. doi:10.1021/jacs.7b02736
